# Altered Intracellular Trafficking as a Mechanism for Prolonged Duration of G Protein-Coupled Receptor Activation

**DOI:** 10.1021/jacs.6c02192

**Published:** 2026-05-20

**Authors:** Tae Wook Kim, Elliot J. Gerrard, Jeeeun Shin, Thomas J. Gardella, Denise Wootten, Patrick M. Sexton, Brian P. Cary, Samuel H. Gellman

**Affiliations:** † Department of Chemistry, University of Wisconsin − Madison, Madison, Wisconsin 53706, United States; ‡ Drug Discovery Biology Theme, Monash Institute of Pharmaceutical Sciences, 2541 Monash University, Parkville 3052, VIC, Australia; § ARC Centre for Cryo-Electron Microscopy of Membrane Proteins, Monash Institute of Pharmaceutical Sciences, 2541 Monash University, Parkville 3052, VIC, Australia; ∥ Endocrine Unit, Massachusetts General Hospital and Harvard Medical School, Boston, Massachusetts 02114, United States

## Abstract

G protein-coupled receptors (GPCRs) mediate information transfer to cells from the surrounding environment. In most cases, signaling is initiated or amplified when the receptor binds to an agonist, an event that alters the conformational profile of the receptor. Signal transduction results from interaction between the agonist-receptor complex and cytosolic partners such as G proteins, GPCR kinases (GRKs), and β-arrestins. Changes in agonist structure can lead to “signal bias”, i.e., changes in the relative strength of signaling involving different partners. Some GPCRs, including those activated by long peptide hormones, continue to signal after internalization. In these cases, changes in agonist structure can lead to changes in the relative extent of signaling from different sites, e.g., cell surface vs endosomes (“location bias”). Many GPCRs are targets of approved drugs or drug candidates, and tuning signal bias and/or location bias is widely considered to be important for optimizing therapeutic profiles. Here we report another mechanism of modulating outcome via agonist modification: alteration of intracellular trafficking. The synthetic peptide agonist designated SPT, which contains five β-amino acid residues, was previously shown to activate the parathyroid hormone receptor-1 (PTH1R) and cause prolonged signaling in mice by an unknown mechanism. The SPT-PTH1R complex continues to stimulate cAMP production after internalization. We now find that the SPT-PTH1R complex impairs the sorting of early endosomes into recycling endosomes relative to the receptor complexed to the drug teriparatide. These findings suggest that altering intracellular GPCR trafficking patterns represents an unappreciated strategy for achieving prolonged action *in vivo*.

## Graphical abstract

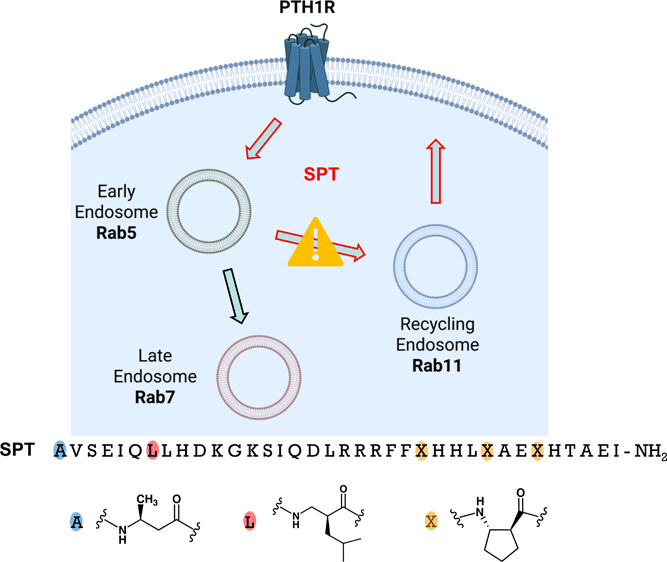

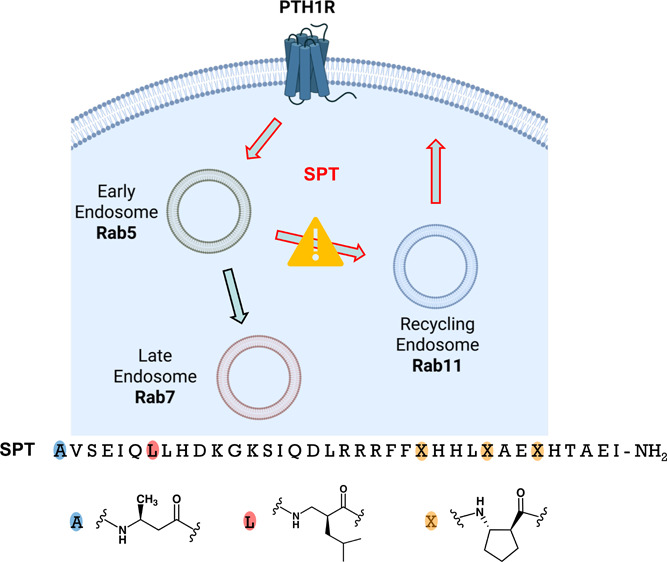

## Introduction

G protein-coupled receptors (GPCRs) respond to a broad range of molecular messages that arrive at cell surfaces. Information encoded in agonists is relayed by the cognate receptor(s) to the cytoplasm. For class B1 GPCRs, the natural agonists are long polypeptide hormones such as parathyroid hormone (PTH; 84 residues) or glucagon (29 residues). Receptors in this class feature a large extracellular domain (ECD), which provides part of the binding surface for the agonists. In most cases, the agonist N-terminus engages the core of the receptor transmembrane domain (TMD) to initiate signal transduction.^1–4^ Many class B1 GPCRs are targets of human drugs, including teriparatide,^5^ which activates the parathyroid hormone receptor-1 (PTH1R), and semaglutide,^6^ which activates the glucagon-like peptide-1 receptor (GLP-1R). Teriparatide is used to treat osteoporosis and comprises the first 34 residues of PTH (PTH(1–34)). Abaloparatide (ABL), another osteoporosis drug, is derived from the first 34 residues of parathyroid hormone-related protein (PTHrP).^7,8^

Changes in agonist structure can cause variations in the signal transduced by a GPCR. One source of variation emerges from the multiplicity of cytosolic proteins with which GPCRs interact, including G proteins, β-arrestins, and GPCR kinases (GRKs). An agonist displays “signal bias” if the pattern of activation along distinct pathways differs from the pattern displayed by a reference agonist.^9–11^ For example, replacement of a single α-amino acid residue with a homologous β-amino acid residue (native side chain; backbone extended by one CH_2_ unit) near the N-terminus of PTH(1–34) or ABL can result in bias away from β-arrestin recruitment relative to stimulation of intracellular cAMP production in cell-based assays.^12,13^ Agonists that display signal bias have been widely discussed as a potential source of drug candidates, because the desired therapeutic effect may depend upon only one signaling pathway, and activation of other pathways might be deleterious.^14–16^

A second form of functional variation among agonists has been described as “location bias”.^17–19^ Activation of many GPCRs leads to receptor internalization and a concomitant termination of signaling.^20^ For some receptors, however, signaling can continue after the receptor has been translocated to endosomes. The PTH1R was among the first receptors for which evidence of intracellular signaling was reported, in this case manifested as production of cAMP within the cytosol.^21,22^ Different locations of cAMP production mediated by the PTH1R (plasma membrane vs cytosol) result in different physiological responses.^12^ Experiments leading to this conclusion relied on an unnatural agonist, the diastereomer of PTH(1–34) with d-Leu at position 7, which stimulates cAMP production via the PTH1R but is defective in terms of receptor internalization. Defective internalization was correlated with diminished β-arrestin recruitment induced by the d-Leu-7 diastereomer relative to PTH(1–34). Thus, the unnatural agonist was biased away from β-arrestin recruitment relative to stimulating cAMP production (signal bias).^12^

Here we report that agonist-induced alteration of intracellular receptor trafficking, which is distinct from signal bias or location bias, can lead to prolonged receptor activation. We recently reported a backbone-modified PTH1R agonist, designated SPT here, that induces a prolonged pharmacodynamic effect *in vivo*.^23^ SPT (“peptide 14” in ref 23) is derived from the 36 N-terminal residues of parathyroid hormone-related polypeptide (PTHrP), with five α-amino acid residues replaced by β-amino acid residues and His5 replaced by Ile (Figure 1A).^23^ Peptides such as PTHrP(1–36) and PTH(1–34) are very potent agonists of the PTH1R, as measured by their ability to stimulate cAMP production in cellular assays.^24^ PTH(1–34) and PTHrP(1–36), however, manifest different spatiotemporal patterns of receptor activation in cells. PTHrP(1–36) causes transient receptor activation at the cell surface; activation is terminated upon PTH1R internalization. In contrast, cAMP production induced by PTH(1–34) continues after receptor internalization,^21,25–27^ which has been attributed to the ability of this peptide to stabilize a complex in which the Gβγ heterodimer and β-arrestin are bound to the receptor.^22^ cAMP production is terminated when these intracellular partners are replaced by the retromer complex, which mediates return of the receptor to the cell surface, presumably by way of recycling endosomes.^28^

**Figure 1:**
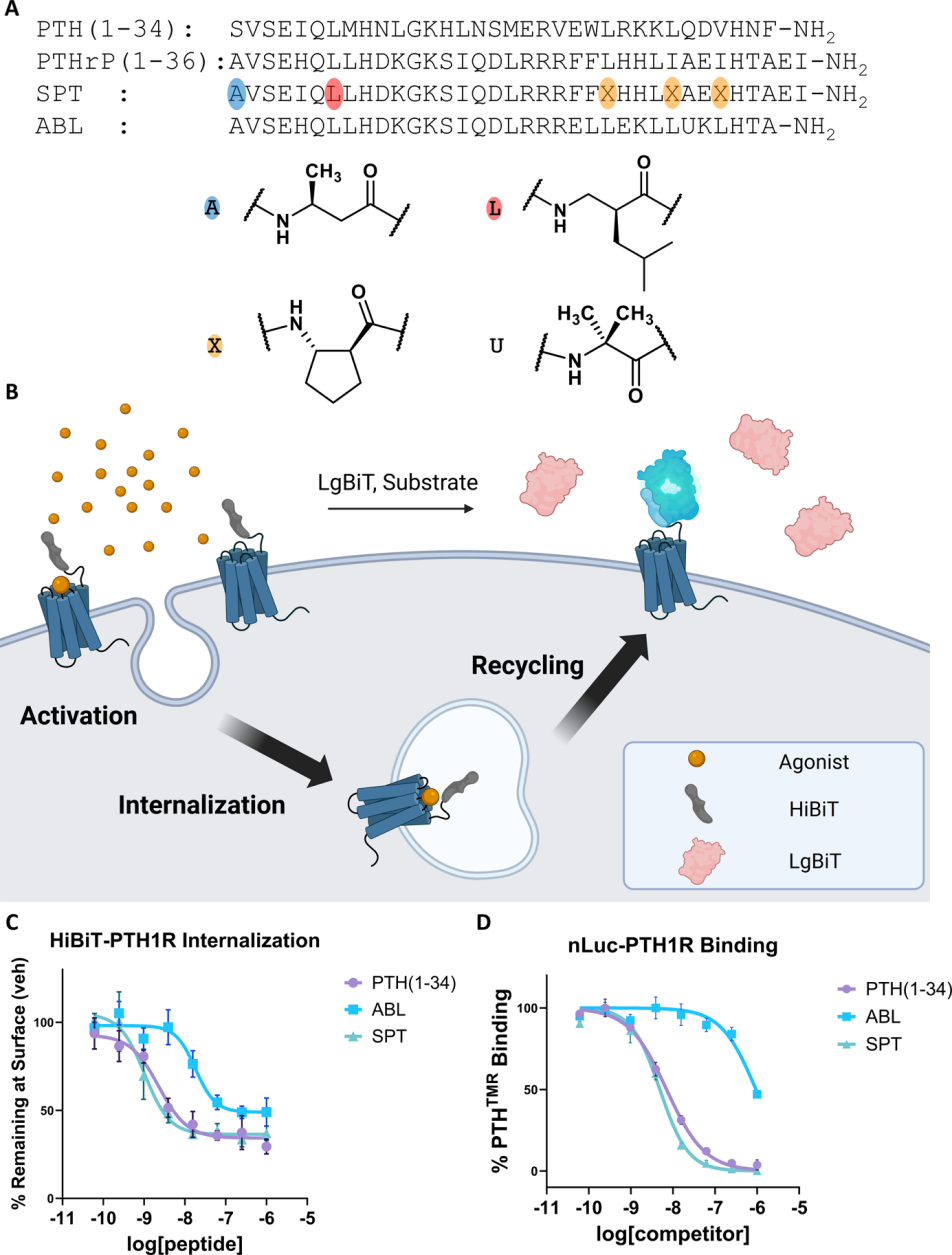
(A) Amino acid sequences of PTH(1–34), PTHrP(1–36), SPT, and ABL, and the structures of non-proteinogenic amino acid residues. (B) Cartoon schematic of HiBiT-PTH1R internalization assay. Stimulation of cells expressing HiBiT-PTH1R with PTH1R agonists results in receptor internalization and concomitant reduction of available HiBiT tag at the cell surface. Subsequent addition of membrane-impermeable LgBiT protein and the nLuc substrate leads to luminescence that is dependent on the amount of HiBiT-PTH1R remaining at the surface. Lower luminescence signifies a higher degree of internalization. (C) Comparative concentration–response data for HiBiT-PTH1R internalization stimulated by PTH(1–34), ABL, or SPT. Data set represents mean ± SD *n* = 3. (D) Data for displacement of PTH(1–34)-K35­(TMR) from nLuc-PTH1R upon addition of PTH(1–34), ABL, or SPT. Data set represents mean ± sem. *n* = 3. The cartoons were generated using Biorender.

The data reported here indicate that SPT induces PTH1R internalization but hinders sorting of early endosomes into recycling endosomes and the subsequent reappearance of the receptor at the cell surface. SPT-induced lingering of the activated PTH1R in the early endosome compartment may explain why SPT causes an *in vivo* calcemic effect that is much prolonged relative to the calcemic effect caused by PTH(1–34).^23^ These findings are important because strategies to achieve prolonged pharmacodynamic effects have been widely pursued to optimize clinical performance of peptide hormone analogues, especially those active at the GLP-1R (e.g., liraglutide, semaglutide, and tirzepatide). In these cases, the duration of action is extended by modifying the peptide agonist with a fatty acid-like appendage, which induces binding to serum albumin and thereby hinders renal excretion.^6,29^ Extending the duration of action via alteration of intracellular receptor trafficking represents a conceptually novel approach.

Current efforts to develop new drugs for type 2 diabetes and obesity via activation of the GLP-1R and/or related class B1 GPCRs have highlighted the importance of receptor trafficking in determining the clinical performance.^30,31^ This perspective has motivated efforts to manipulate the extent of the receptor internalization. Our findings suggest that broadening these considerations to include post-internalization trafficking could reveal new approaches for tailoring clinical performance.

## Results and Discussion

### Agonist-Induced Internalization of HiBiT-PTH1R

The experimental program described here was motivated by our previous finding that injection of SPT induced an increase in calcium in the mouse bloodstream over a substantially longer period relative to the increase in calcium induced by PTH(1–34).^23^ Measurements using UMR-106 cells, which are derived from rat osteosarcoma tissue and natively express the PTH1R, showed prolonged signaling as detected by cAMP production after stimulation by SPT compared to stimulation by PTH(1–34) (Figure S1A). SPT was ∼10-fold more potent in these assays (i.e., EC_50_ was ∼10-fold lower for SPT) relative to PTH(1–34) (Figure S1B); each agonist was used at a concentration near the EC_50_. These observations are consistent with the prolonged calcemic effect induced *in vivo* by SPT.

We compared SPT, PTH(1–34), and ABL in terms of their ability to induce internalization of the PTH1R in HEK293 cells engineered to express the receptor. To measure receptor loss from the cell surface, we used a nanoluciferase (nLuc) complementation strategy that was previously employed for two class A GPCRs.^32,33^ HiBiT, an 11-mer peptide derived from the C-terminus of nLuc, binds with high affinity (*K*_D_ = 0.7 nM) to the larger LgBiT fragment of nLuc (18 kDa). Formation of the HiBiT-LgBiT complex generates a functional luciferase, and this complementation can be readily detected by luminescence. We fused HiBiT to the N-terminus of the PTH1R (HiBiT-PTH1R). In the basal cellular state, most receptor molecules reside in the plasma membrane,^27^ and this population can be detected on HEK293 cells transfected to express HiBiT-PTH1R by luminescence after addition of LgBiT and luciferase substrate to the growth medium. Association of LgBit with the HiBit module in the N-terminally modified PTH1R generates a species very similar to nLuc-PTH1R, which has previously been shown to manifest activation behavior similar to that of unmodified PTH1R.^34,35^ LgBiT should not cross the plasma membrane,^29^ which allows HiBiT-PTH1R internalization to be monitored via agonist-induced reduction in luminescence (Figure 1B).

Luminescence was measured after HEK293 cells expressing HiBiT-PTH1R had been stimulated with PTH(1–34) for 30 min by adding LgBiT protein and the nLuc substrate. As expected, higher concentrations of PTH(1–34) resulted in lower luminescence (Figure 1C). The EC_50_ value derived from the concentration–response data (2.2 ± 0.5 nM) was consistent with an earlier report involving a commercially available assay that measures PTH1R recruitment to endosomes.^35^ Another previous study detected PTH1R internalization using a pH-sensitive derivative of green fluorescent protein (GFP^pHs^); a decline in GFP^pHs^ fluorescence was interpreted as internalization into acidic endosomes.^36^ In this case, an EC_50_ value was not determined.

We used the nLuc complementation assay to compare the potencies of PTH(1–34), ABL, and SPT for inducing receptor internalization (Figure 1C). Modest variations were observed (Table 1), with SPT being the most potent and ABL being the least potent in causing receptor removal from the cell surface. A previous comparison of PTH(1–34) and ABL with an analysis based on GFP^pHs^ fluorescence did not detect any difference in internalization activity.^36^ The assay we employed may be more sensitive than the assay used in the previous study, which would explain our detection of a small difference in internalization potencies between PTH(1–34) and ABL. Alternatively, these modest variations may reflect differences in the assay formats.

**Table 1:** Comparisons of Binding and Internalization Data for PTH­(1-34), ABL, and SPT^a^

	**Binding IC**_**50**_ **(nM)**	**Internalization EC**_**50**_ **(nM)**
**PTH(1–34)**	7.1 ± 0.6	2.2 ± 0.5
**ABL**	940 ± 100^b^	18 ± 4^c^
**SPT**	4.8 ± 0.5^d^	1.0 ± 0.1^d^

a *Binding IC*_50_: measurements with nLuc-PTH1R were conducted by treating HEK293 cells stably expressing the nLuc-PTH1R with the tracer PTH(1–34)-K35^TMR^ at a constant concentration and unlabeled PTH(1–34), ABL, or SPT at varying concentrations. *Internalization EC*_50_ *:* measurements with HiBiT-PTH1R were conducted by treating HEK293 cells transiently transfected with HiBiT-PTH1R with varying concentrations of PTH(1–34), ABL, or SPT in the presence of cycloheximide and then monitoring the luminescence response following the addition of LgBiT protein and the nLuc substrate, furimazine. Each value represents mean ± sem from *n* = 3 independent experiments. One-way ANOVA followed by Dunnett’s multiple comparisons test were used to calculate the significance of values relative to PTH(1–34) for EC_50_ values.

b *p* < 0.0001.

c *p* < 0.01.

d Not significant.

As a complement to the internalization data, we compared affinities of the three peptides for the PTH1R with a recently reported competition assay based on displacement of a fluorescently labeled derivative of PTH(1–34) from the receptor.^34^ PTH(1–34) and SPT displayed similar IC_50_ values in this assay, while IC_50_ for ABL was >100-fold higher, indicating that ABL was less effective at displacing the tracer relative to PTH(1–34) or SPT (Figure 1D). Comparison of internalization and binding data suggested that differences in affinity for the receptor may contribute to but cannot fully explain differences in the potencies with which SPT, PTH(1–34), and ABL induced disappearance of the PTH1R from the cell surface.

### Intracellular Distribution of the PTH1R after Agonist Simulation Monitored by Microscopy

The modest variation between PTH(1–34) and SPT in potency for inducing receptor internalization does not seem to explain why the calcemic effect induced by SPT in mice is of substantially longer duration than the calcemic effect induced by PTH(1–34).^23^ We therefore considered the possibility that PTH(1–34) and SPT cause differential trafficking of agonist-PTH1R complexes within cells.^37^ Recently, two natural agonists of the κ opioid receptor (KOR), dynorphins A and B, were reported to cause different proportions of receptor degradation vs recycling, which suggests divergent intracellular trafficking of KOR-dynorphin complexes.^38^ We therefore explored the fate of the PTH1R expressed in HEK293 cells after activation by PTH(1–34), SPT or ABL.

To monitor the intracellular location of the PTH1R via fluorescence microscopy, we fused a fluorescent protein to the receptor N-terminus to generate eGFP-PTH1R. To identify intracellular vesicles, we used Rab proteins^39^ fused to mCherry: Rab5 for early endosomes, Rab7 for late endosomes, or Rab11 for recycling endosomes. Receptors in late endosomes are targeted for destruction, while receptors in recycling endosomes will reappear at the plasma membrane.^40^ DNA encoding eGFP-PTH1R and DNA encoding one of the mCherry-Rab proteins were transiently transfected into HEK293 cells. After 30 min of incubation with agonist, the cells were fixed with formalin and examined via confocal microscopy.

Micrographs of cells expressing eGFP-PTH1R and mCherry-Rab5 showed strong alignment in the fluorescent intensities after simulation with each of the three peptides, indicating that the PTH1R is translocated to early endosomes after activation with each agonist (Figure 2A, Figure S1C,D). These results are consistent with previous findings that each of these peptides causes recruitment of β-arrestins to the PTH1R,^23,41^ which is a prerequisite for clathrin-mediated endocytosis of this receptor.^20^ In contrast, colocalization of eGFP-PTH1R with mCherry-Rab7 was not detected via microscopy after stimulation with any of the peptides (Figure 2B, Figure S1E,F).

**Figure 2:**
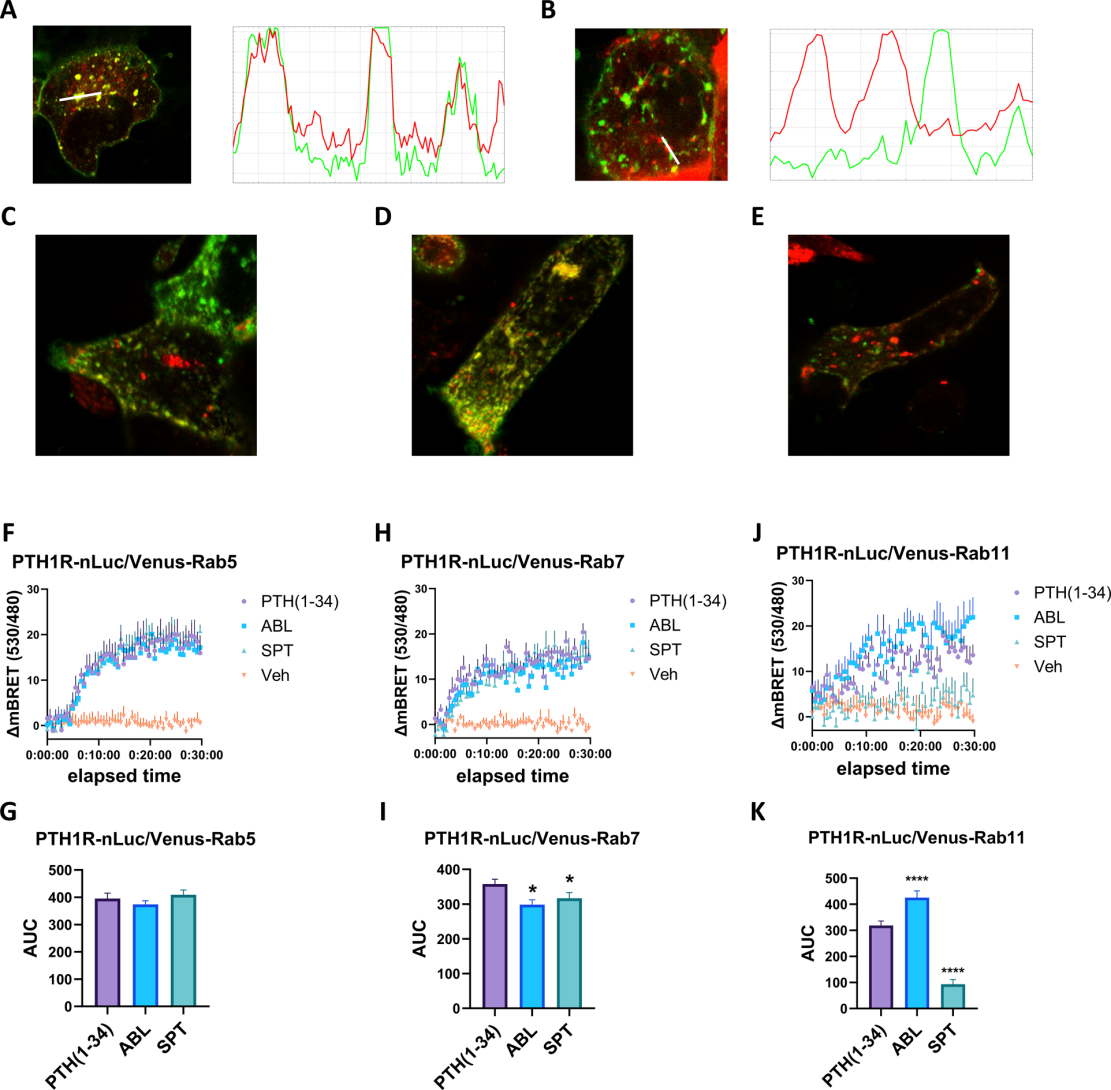
(A, B) Micrographs and fluorescence plots representing the location of PTH1R and Rab5 or Rab7 after stimulation by PTH(1–34). Cells expressing eGFP-PTH1R and either Rab5-mCherry (A) or Rab7-mCherry (B) were stimulated by 100 nM PTH(1–34) for 30 min, fixed, and then observed under a confocal microscope via 588 and 561 nm emission channels. Fluorescence plots represent the relative fluorescence of eGFP (green) and mCherry (red) along the white line in the micrograph. (C-E) Micrographs showing the location of PTH1R and Rab11 after stimulation by PTH(1–34), ABL, or SPT. Cells expressing eGFP-PTH1R and Rab11-mCherry were stimulated by 100 nM PTH(1–34) (C), ABL (D), or SPT (E) for 30 min, fixed,and then observed under a confocal microscope via 588 and 561 nm emission channels. (F, H, J) Kinetic BRET response curves of PTH1R-nLuc/Venus-Rab­(5/7/11) following stimulation. Cells expressing PTH1R-nLuc and Venus-Rab5 (F), Venus-Rab7 (H), or Venus-Rab11 (J) were stimulated with 1 μM agonist, and the BRET response was measured over 30 min. Data set represents mean + sem. n = 3. (G, I, K) AUC values of the kinetic response curve calculated from (F, H, J), respectively. One-way ANOVA followed by Dunnett’s multiple comparisons test were used to calculate the significance of values relative to PTH(1–34). (*: *p* < 0.05, ****: *p* < 0.0001).

PTH­(1–34), ABL, and SPT diverged in trafficking of the PTH1R to recycling endosomes marked by mCherry-Rab11 (Figure 2C-E). Both PTH(1–34) and ABL induced colocalization of eGFP-PTH1R with mCherry-Rab11, while SPT did not. Collectively, these data suggest that agonist identity determines the intracellular trafficking pattern of the PTH1R. Relative to PTH(1–34), SPT impedes sorting of early endosomes containing the PTH1R to recycling endosomes.

### PTH1R Trafficking Monitored with BRET Assays

To complement qualitative observations from fluorescence microscopy, we undertook measurements using bioluminescence resonance energy transfer (BRET) between PTH1R fused at its C-terminus to nLuc (BRET donor) and a Rab protein fused to the fluorescent protein Venus (BRET acceptor). A similar design has been used to characterize the recruitment of Venus-labeled β-arrestins to a PTH1R fused to RLuc8 at the C-terminus.^42^ Analogous BRET assays have been used to characterize agonist-induced trafficking of two other class B1 GPCRs, the GLP-1R and the gastric inhibitory polypeptide receptor (GIPR).^43^ We observed that PTH(1–34), ABL or SPT (1 μM for each agonist) elicited a comparable BRET response from cells coexpressing PTH1R-nLuc and Venus-Rab 5 (Figure 2F,G). These data are consistent with the microscopy study in suggesting that stimulation of the receptor with each agonist induces translocation to early endosomes.

BRET data from HEK293 cells coexpressing PTH1R-nLuc and Venus-Rab 7 indicated that all three peptides cause the receptor to be found in late endosomes, which is not consistent with the microscopy studies (Figure 2H,I). This contrast may indicate that the BRET-based assay is more sensitive than fluorescence microscopy for detecting colocalization. Alternatively, this contrast between assays could have arisen because different peptide amounts were used, 0.1 μM for the microscopy studies vs 1.0 μM for the BRET studies, but both peptide concentrations should have been fully saturating in terms of internalization. Differences among PTH(1–34), ABL and SPT were modest in the receptor-Rab 7 colocalization assay, as indicated by area-under-the-curve (AUC) analysis. The Rab7 BRET signal showed a steady increase throughout the 30 min measurement period for each agonist. This behavior contrasted with the Rab5 assays, in which the BRET signal reached a plateau within 30 min for all three agonists.

Receptor-Rab 11 BRET measurements revealed substantial differences among the three peptides. PTH(1–34) and ABL were very effective at causing the PTH1R to appear in recycling endosomes, with ABL causing a moderately higher level of receptor-Rab 11 colocalization than did PTH(1–34). In contrast, SPT caused very little Rab11 BRET signal (Figure 2J,K). This result supports the conclusion drawn from microscopy data: PTH1R bound to SPT interferes with sorting of early to recycling endosomes, which suggests that SPT enables prolonged internalization of the receptor.

### Receptor Recycling after Agonist Stimulation

Based on the observation that SPT led to lower PTH1R levels in recycling endosomes relative to PTH(1–34) or ABL, we predicted that the receptor should reappear at the cell surface more rapidly after cells were stimulated with PTH(1–34) or ABL relative to stimulation with SPT. We tested this prediction with experiments involving HEK293 cells expressing HiBiT-PTH1R. These cells were treated with cycloheximide and exposed to agonist for 30 min. Excess agonist was then washed away, and the cells were treated with the antagonist PTH(3–34), which is comparable to PTH(1–34) in terms of affinity for the PTH1R^34^ but does not cause G protein activation or β-arrestin recruitment.^44^ This antagonist was intended to deter rebinding of residual agonist to the PTH1R. In addition, the cells were treated with cycloheximide to prevent HiBiT-PTH1R synthesis during the experiment. The resulting luminescence curves were normalized to the response from vehicle-treated cells, which represents the receptor level at the cell surface in the absence of agonist stimulation.

Cells treated with ABL showed rapid reappearance of HiBiT-PTH1R at the cell surface; slower receptor reappearance was observed for cells treated with PTH(1–34) (Figure 3A,B). These observations are consistent with data previously reported for comparison of ABL and PTH(1–34) via GFP^pHs^ fluorescence.^36^ In contrast to the other two agonists, SPT did not cause detectable HiBiT-PTH1R reappearance on the cell surface during the period of observation. Diminished receptor recycling for a different long-acting agonist, LA-PTH, has previously been reported on the basis of GFP^pHs^ fluorescence measurements.^44^ The lack of receptor recycling after cell stimulation by SPT is consistent with data presented above indicating that this backbone-modified agonist alters intracellular trafficking relative to ABL or PTH(1–34), with the SPT-receptor complex impairing sorting of early endosomes to recycling endosomes.

**Figure 3:**
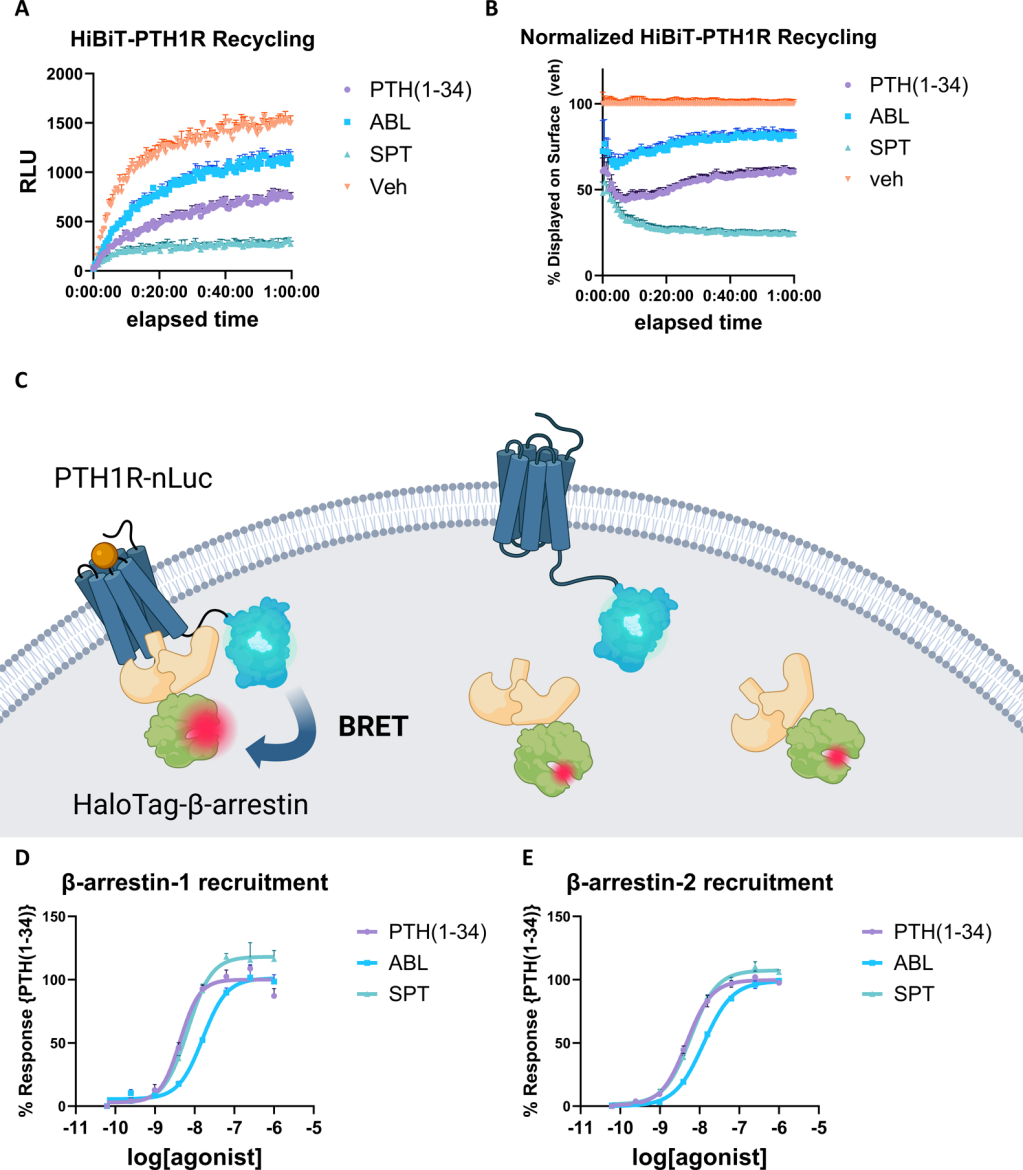
(A) Kinetic luminescence curve of HiBiT-PTH1R recycling. Cells expressing HiBiT-PTH1R were stimulated with 100 nM PTH(1–34), ABL, SPT, or vehicle for 30 min then washed out in the presence of PTH1R antagonist PTH(3–34), LgBiT protein, and the nLuc substrate. Luminescence from HiBiT+LgBiT was measured over the course of 1 h. (B). Normalized kinetic curves. The luminescence values from (A) were normalized to the values obtained from vehicle-treated cells. (C) Cartoon schematic of total PTH1R-nLuc/HaloTag-β-arrestin recruitment. Recruitment of β-arrestins to the PTH1R results in BRET from nLuc to the fluorescently labeled HaloTag. (D, E) Concentration–response curve for recruitment of β-arrestin-1 or -2 to PTH1R. Cells expressing PTH1R-nLuc and HaloTag-β-arrestin-1 (D) or HaloTag-β-arrestin-2 (E) were stimulated with PTH(1–34), ABL, or SPT for 30 min. The BRET responses at the 30 min mark were used to generate the concentration–response curves. Data set represents mean + sem. *n* = 3. The cartoons were generated using Biorender.

### Location Specific β-Arrestin Recruitment to the PTH1R

β-Arrestins mediate the internalization of many GPCRs.^45–47^ For the PTH1R, β-arrestin binding is essential for intracellular cAMP production; displacement of β-arrestin terminates cAMP signaling and enables recycling to the plasma membrane.^28^ To explore whether the alterations in intracellular trafficking revealed by the experiments described above for SPT relative to PTH(1–34) or ABL could arise from a distinctive mode of β-arrestin engagement with the receptor-SPT complex, we conducted assays for global recruitment of β-arrestins to the PTH1R using a well-precedented BRET-based strategy by pairing a previously reported PTH1R-nLuc fusion protein,^42^ which was also used in the Rab colocalization studies, with versions of β-arrestin-1 or β-arrestin-2 fused to HaloTag^48^ (Figure 3C). When HEK293 cells expressing PTH1R-nLuc and one of the β-arrestin fusion proteins were exposed to any of the three agonists, a robust, concentration-dependent BRET signal was observed (Figure 3 D,E). ABL manifested a moderately lower potency for recruitment of either β-arrestin-1 or β-arrestin-2 to the PTH1R relative to PTH(1–34) or SPT (Table 2, *p* < 0.005). One previous report indicated slightly more potent β-arrestin recruitment for ABL relative to PTH(1–34).^41^ This study employed a commercial luminescence-based assay involving enzyme fragment complementation CHO-K1 cells, and it is not clear which β-arrestin was used. Another recent study employed BRET to monitor recruitment of β-arrestin in CHO-FlpIn cells expressing a PTH1R-luciferase fusion and either β-arrestin-1 or β-arrestin-2 fused to the venus fluorescent protein.^13^ In these studies, ABL was slightly less potent than PTH(1–34). Collectively, these two studies indicated that PTH(1–34) and ABL are similar in their recruitment potencies, and our data support this conclusion.

**Table 2:** Comparisons of EC_50_ and *E*_max_ Data for PTH­(1-34) ABL, and SPT for β-Arrestin Recruitment^a^

	**EC**_**50**_ **(nM)**		***E***_**max**_ **(% PTH(1–34))**	
	**β-arrestin-1**	**β-arrestin-2**	**β-arrestin-1**	**β-arrestin-2**
**PTH(1–34)**	4.3 ± 0.5	4.7 ± 0.3	100 ± 3	99 ± 1
**ABL**	16 ± 2^b^	12 ± 1^c^	101 ± 4^e^	98 ± 1^e^
**SPT**	6.7 ± 1^e^	5.9 ± 0.4^e^	118 ± 3^d^	107 ± 2^d^

a The recruitment of β-arrestin-1 or β-arrestin-2 was measured using HEK293 cells transiently transfected to express the PTH1R-nLuc and HaloTag-β-arrestin-1 or HaloTag-β-arrestin-2 constructs. The cells were treated with PTH(1–34), ABL, or SPT at varying concentrations in the presence of the nLuc substrate, furimazine, and the BRET response was monitored. Each value is the mean ± sem from *n* = 3 independent experiments. One-way ANOVA followed by Dunnett’s multiple comparisons test were used to calculate the significance of values relative to PTH(1–34) for the values.

b *p* < 0.01.

c *p* < 0.001.

d *p* < 0.005.

e Not significant.

SPT was very similar to PTH(1–34) in terms of potency for stimulating recruitment of either β-arrestin-1 or β-arrestin-2 to the PTH1R (Figure 3D,E, Table 2). However, SPT displayed a higher maximum than did PTH(1–34) in each assay. Comparable observations were previously reported in an independent β-arrestin recruitment assay (different cell line, different BRET donor/acceptor pair).^23^ The consistent pattern in β-arrestin recruitment maxima in the two assays suggests that the mode of engagement between the receptor and each β-arrestin may differ for these two agonists in terms of geometry and/or stability.

The nature of the interaction between the receptor and a β-arrestin has been suggested to influence time scale of GPCR recycling after internalization; these interactions involve the C-terminal domain of the receptor.^22,28,49^ We hypothesized that a distinct mode of PTH1R-β-arrestin association at the endosomal membrane mediated by SPT, relative to the PTH1R-β-arrestin association mediated by PTH(1–34), could hinder the sorting of early endosomes that harbor the receptor and ultimately slow the rate of receptor recycling. To probe this possibility, we developed two new BRET assays based on a novel design that allowed location-specific assessment of recruitment of β-arrestin-1 or β-arrestin-2 recruitment to the PTH1R. One assay reported on β-arrestin recruitment to the receptor in the endosomal membrane, and the other assay reported on β-arrestin recruitment to the receptor in the plasma membrane. Site-specificity was achieved by using a split luciferase as the BRET donor. One component of the split luciferase was fused to the PTH1R C-terminus, and the other luciferase component was fused to a unit that enforces localization to either the plasma membrane (CaaX)^50^ or the early endosomal membrane (EndoFin).^51^ The β-arrestin-HaloTag fusion proteins described above were employed as the BRET acceptors (Figure 4A,B).

**Figure 4:**
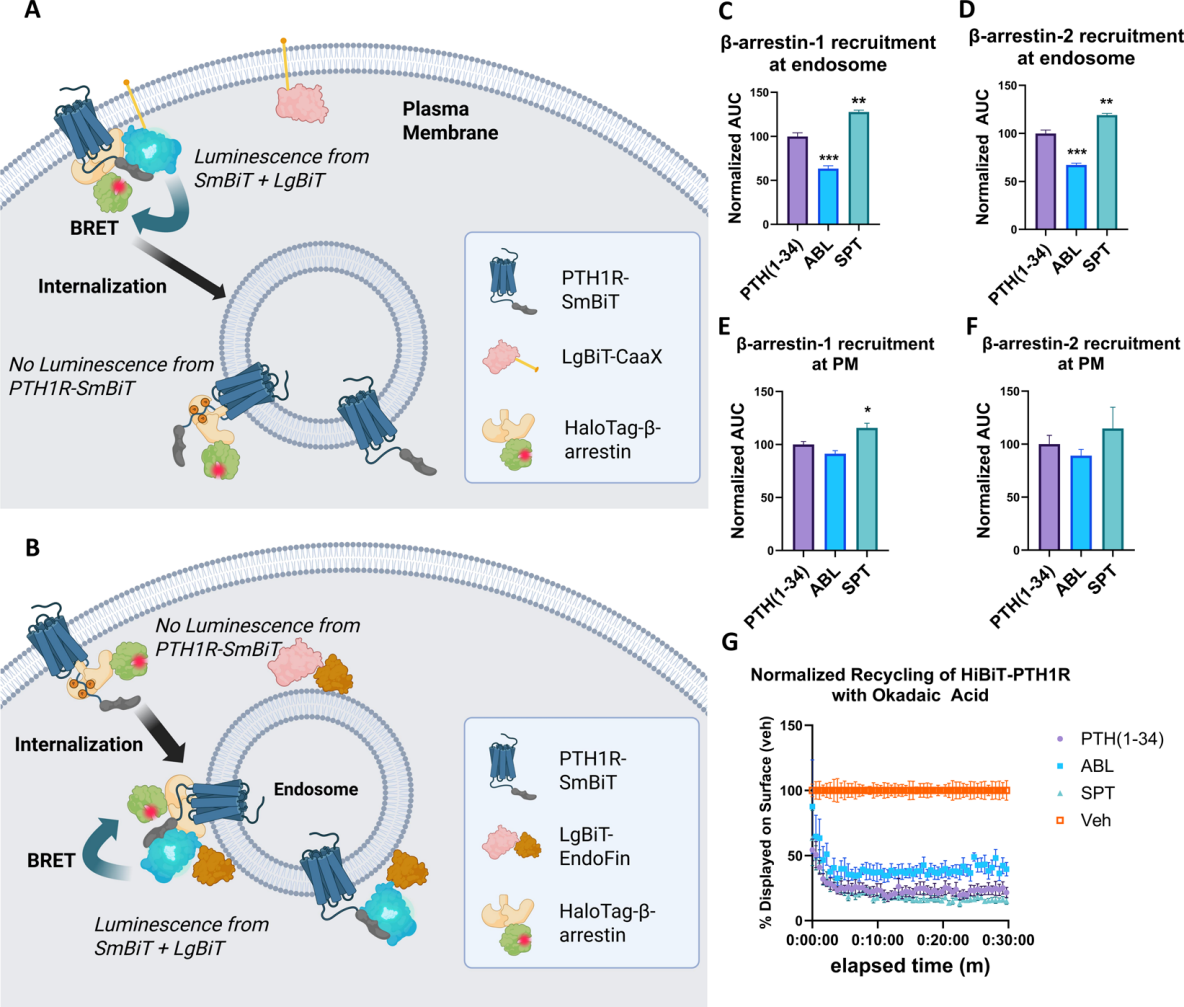
(A, B) Cartoon schematic of β-arrestin recruitment at the plasma membrane (A) or the endosome (B). For the endosome-specific β-arrestin recruitment, PTH1R-SmBiT, HaloTag-β-arrestin, and LgBiT-EndoFin were coexpressed. At the basal state, PTH1R-SmBiT resides at the cell surface, does not generate luminescence and therefore does not produce a BRET response even when HaloTag-β-arrestin is bound. When PTH1R-SmBiT internalizes to the early endosomes, the SmBiT tag complements with the LgBiT and produces luminescence. When HaloTag-β-arrestin is bound to PTH1R at the endosomes, the SmBiT-LgBiT (NanoBiT) produces a BRET response. (C−F) Bar plots of AUC values generated from the BRET response between PTH1R-SmBiT and HaloTag-β-arrestin when PTH1R-SmBiT is at the endosomes (C, D) or at the plasma membrane (E, F). Data set represents the mean + sem. *n* = 3. One-way ANOVA followed by Dunnett’s multiple comparisons test were used to calculate the significance of values relative to PTH(1–34). (*: *p* < 0.05, **: *p* < 0.01, ***: *p* < 0.001) (F) Normalized kinetic response curve of HiBiT-PTH1R recycling. Conditions were identical to those for Figure 3B except for the presence of 1 μM okadaic acid. Data set represents mean ± sem. *n* = 3. The cartoons were generated using Biorender.

These location-specific β-arrestin recruitment assays used the SmBiT+LgBiT pair to generate luciferase activity. In contrast to the 11-mer HiBiT peptide, the 11-mer SmBiT peptide has a relatively low affinity (*K*_D_ = 190 μM) for the LgBiT component.^52^ Therefore, SmBiT binding to LgBiT is unlikely to drive spontaneous association of two proteins that bear these components. If those two proteins are brought into proximity, however, SmBiT+LgBiT complementation can occur to create a functional luciferase, which provides a way to detect the proximity of the two proteins. Our assays paired PTH1R-SmBiT^53^ with either a LgBiT-CaaX fusion protein, to detect β-arrestin recruitment at the plasma membrane, or a LgBiT-EndoFin fusion protein, to detect β-arrestin recruitment at the early endosome membrane.^54,55^

Following the stimulation of cells expressing PTH1R-SmBiT, LgBiT-EndoFin, and HaloTag-β-arrestin with a saturating level of PTH(1–34) (1 μM), β-arrestin-1 or β-arrestin-2 was robustly recruited to receptor in the endosomal membrane (Figure 4C,D). SPT, at 1 μM, was markedly more effective than PTH(1–34) at recruiting each β-arrestin to the PTH1R in the endosomal membrane. This difference parallels a trend toward higher *E*_max_ values for SPT in the global β-arrestin recruitment assays, but the difference between the peptides in β-arrestin recruitment to the endosomal membrane contrasts with the similar potencies of SPT and PTH(1–34) in the global β-arrestin recruitment assays (Figure 3). ABL was slightly less potent than PTH(1–34) in our global β-arrestin recruitment assays, and at 1 μM, ABL had a similar maximal response for global recruitment. Despite these global similarities, ABL was substantially less effective than PTH(1–34) at recruiting each β-arrestin to the PTH1R in the endosomal membrane.

Complementary assays to evaluate β-arrestin recruitment to the PTH1R at the plasma membrane revealed little or no difference among the three peptides. Thus, stimulation with 1 μM of each peptide led to indistinguishable levels of β-arrestin-2 recruitment at the plasma membrane (Figure 4E,F). For β-arrestin-1 recruitment at the plasma membrane, PTH(1–34) and ABL were indistinguishable, but a small enhancement was detected for SPT relative to PTH(1–34). Taken together, the complementary site-specific β-arrestin recruitment assays highlight agonist-dependent differences in engagement between the PTH1R and β-arrestin that are unique to the endosomal membrane. The higher recruitment of β-arrestins to endosomal PTH1R induced by SPT suggests that the SPT-PTH1R-arrestin complex may be more resistant than the PTH(1–34)-PTH1R-arrestin complex to β-arrestin displacement mediated by the retromer complex during the endosomal sorting process that is required for recycling. On the other hand ABL causes diminished β-arrestin recruitment to endosomal PTH1R, consistent with the greater PTH1R recycling observed for this agonist.

### Effect of Phosphatase Inhibitor on the Recycling Rate

Phosphorylation at intracellular sites of a GPCR, most typically the C-terminal domain, is crucial for optimal β-arrestin binding.^37^ Following internalization, GPCRs are dephosphorylated by phosphatases at the endosome.^56–61^ Resensitization of cells, which presumably reflects recycling of internalized receptor to the plasma membrane, requires dephosphorylation of the receptor.^58^ Access of phosphatases to phosphosites in the receptor requires dissociation of β-arrestins to expose the phosphorylation sequences. We hypothesized that agonist-dependent differences in the time scale of PTH1R recycling (Figure 3B) could arise from differences in β-arrestin engagement with receptor-agonist complexes and the resulting impact on phosphatase activity.

We tested this hypothesis by treating cells expressing HiBiT-PTH1R with okadaic acid, a potent phosphatase inhibitor, and measuring the receptor recycling rate. Under these conditions, very little recycling was observed for cells treated with PTH(1–34), ABL or SPT (Figure 4G). This uniformity contrasted with the significant differences in receptor recycling observed for the three peptides in the absence of okadaic acid (Figure 3B; internalization observed in the absence of okadaic acid suggests that this receptor construct retained competence in terms of β-arrestin recruitment). Thus, the phosphatase inhibitor had little or no effect on PTH1R recycling in response to stimulation with SPT, but the inhibitor suppressed the recycling that resulted from stimulation with PTH(1–34) or ABL. Overall, these observations support the hypothesis that the SPT-PTH1R complex is less susceptible to dephosphorylation in endosomes compared to the receptor after activation by PTH(1–34) or ABL, and that the diminished susceptibility results from prolonged association of β-arrestins with the SPT-PTH1R complex.

Our results contrasted with a previous report that two phosphatase inhibitors, okadaic acid and calyculin A, do not affect the recycling of human PTH1R expressed in HEK293 cells.^59^ However, this prior study differed from our own in two important ways: the previous authors employed bovine PTH(1–34) (vs human PTH(1–34) in our study) and opossum PTH1R (vs human PTH1R in our study). Because of sequence differences between agonists and receptors, it is not clear whether comparable results should be expected.

### Structural Elucidation of a PTH1R-Gαs Heterotrimer Complex Bound to SPT

To try to gain structural insights into the activation of the PTH1R by SPT, we performed single particle cryo-electron (cryo-EM) microscopy studies. Human PTH1R and a Gαs heterotrimer were expressed in insect cells, complexed with SPT, and isolated as previously reported (Figure S2A,B).^62^ Following vitrification and imaging, the complex was reconstructed to a global resolution of 3.21 Å (Figure 5A, Figure S2C), enabling atomic modeling for most residues. As with other cryo-EM structures of class B1 GPCRs,^3^ lower resolution was observed for the extracellular domain, indicating high flexibility relative to the rest of the complex. Because of this lower local resolution, much of the ECD was modeled at only the backbone level. 3D-variability analysis^63^ confirmed high flexibility for the ECD, but the SPT interaction with the receptor TMD was rigid, in contrast with results from some other class B1 GPCR agonists (Video S1).^64,65^ We compared our cryo-EM structure of the full-length PTH1R with the X-ray crystal structure of the PTH1R ECD bound to a peptide comprising residues 16–36 of SPT and an N-terminal Tyr residue (Figure 5B, Figure S3).^23^ As expected, the two models agree well at the backbone level (Cα RMSD < 1 Å). Between the two models, however, there are noteworthy differences in the rotamers for His25 and His26 of SPT, which we attribute to crystallographic contacts in the isolated ECD structure.

**Figure 5:**
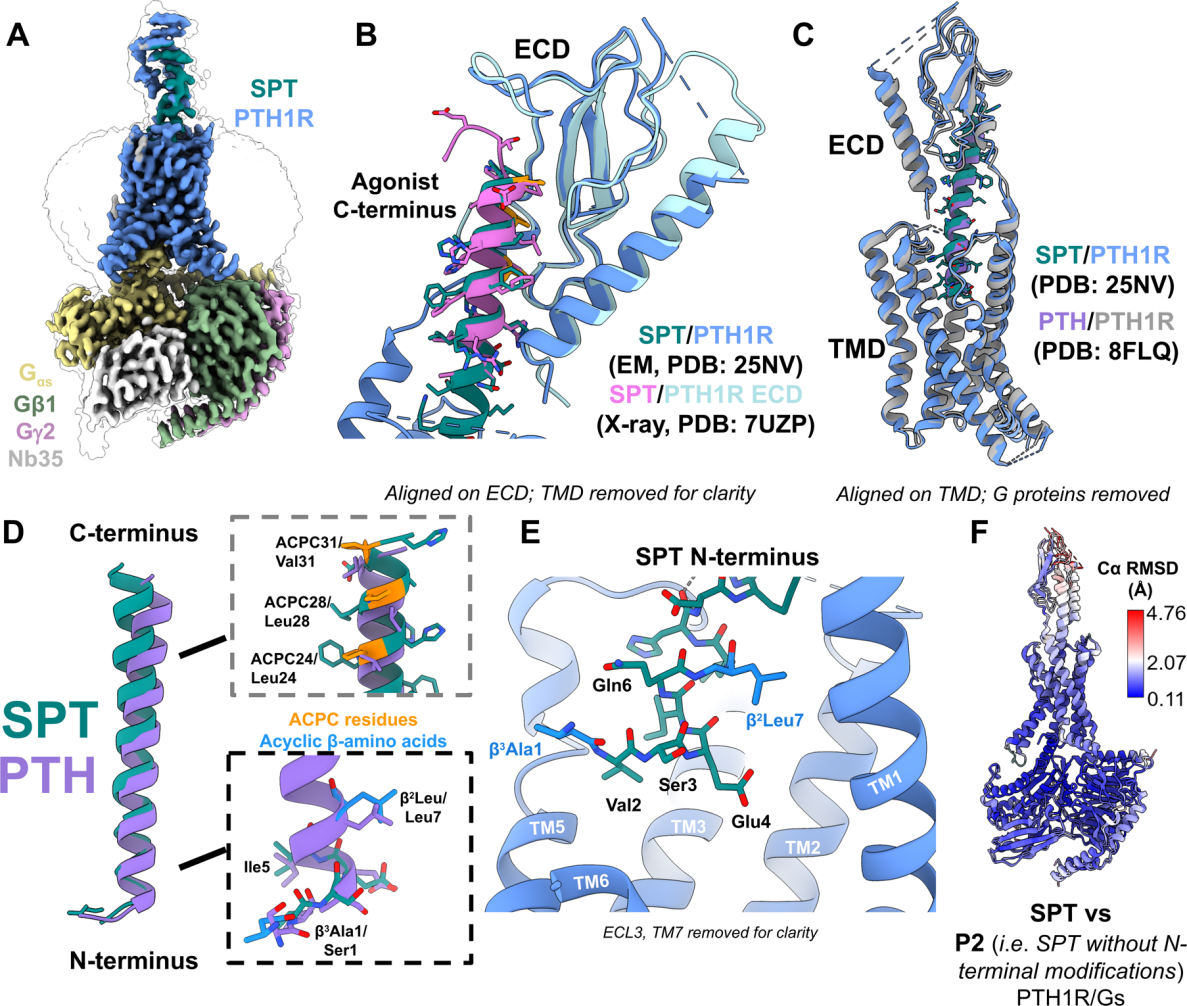
(A) A sharpened cryo-EM map of the complex of PTH1R, SPT, and a G protein heterotrimer. Components are colored according to the keys. The transparent silhouette shows the map at low threshold, to visualize densities for the micelle and ECD. (B) A comparison of the X-ray structure of a PTH1R ECD construct and a derivative of SPT (PDB: 7UZP) and the cryo-EM structure of SPT with the PTH1R. The models were aligned by corresponding ECD residues. (C) An alignment of the structure of PTH(1–34)-bound PTH1R/Gs (PDB: 8FLQ) and the structure of SPT-bound PTH1R/Gs (PDB: 25NV). The alignment was performed on the receptor transmembrane domains. (D) A comparison of the receptor-bound conformations of PTH(1–34) and SPT (when receptor TMDs are aligned). Inset figures bounded by dashed lines show close up images of the backbone modifications included in SPT compared to the native residues in PTH(1–34). (E) A close-up of the TMD orthosteric pocket in the structure of PTH1R and SPT. (F) A comparison of the structures of PTH1R bound to SPT vs P2.

Overall, the structure of the PTH1R bound to SPT is similar to that of the receptor bound to PTH(1–34) (Figure 5C). Previous studies suggested that the (*S*) absolute configuration for β^2^-homoamino acids is optimal for α-helical mimicry,^66,67^ and we observed that (*S*)-β^2^-hLeu7 of SPT adopts a helical conformation to mimic the native, receptor-bound local conformation of Leu7 in PTH(1–34) (Figure 5D,E). Side chains of (*S*)-β^2^-hLeu7 and Leu7 of SPT and PTH(1–34), respectively, occupy a hydrophobic pocket formed by receptor residues Phe184, Leu187, Tyr191, Met441, and Met445 (Figure S4). As observed for Ser1 of PTH(1–34), the (*S*)-β^3^-hAla1 residue of SPT occupies a pocket bounded by receptor residues Trp361, Leu368, Thr427, and Tyr429.

Previous studies with cells expressing a truncated version of the PTH1R lacking the extracellular domain showed that SPT was ∼100-fold more potent in terms of stimulating intracellular cAMP production relative to PTH(1–34).^23^ The analogue of SPT that contained the native PTHrP residues Leu24, Ile28 and Ile31, which are replaced in SPT itself by cyclic β residues, was equipotent to SPT in this assay. These data suggest that the Ala1→(*S*)-β^3^-hAla1 and Leu7→(*S*)-β^2^-hLeu7 replacements and the His5→Ile replacement were wholly responsible for the potency change manifested by SPT at the truncated PTH1R variant. The structural basis for this improved activity, as well as for the altered receptor trafficking activity of SPT, was not readily apparent from the new cryo-EM structure. This observation is not surprising given that previous cryo-EM analyses showed that several PTH1R agonist peptides with a range of activities, including PTH(1–34), PTHrP(1–36) and ABL, adopt similar consensus poses when bound to the PTH1R-Gαs complex.^62^

In parallel to our structural investigations with SPT, we determined a cryo-EM structure of the PTH1R in complex with peptide 2 (P2) from ref 23. P2 is a PTHrP(1–36) analogue that contains the same three ACPC substitutions in its C-terminal region as SPT, but P2 lacks the three N-terminal modifications included in SPT. SPT showed increased affinity and signaling duration relative to P2 at the PTH1R.^23^ We therefore hypothesized that comparison of complexes of SPT or P2 with the PTH1R might reveal a structural basis for the functional differences between the two agonist peptides. However, the SPT+PTH1R and P2+PTH1R structures were very similar (RSMD < 1 Å between the structures containing SPT and P2, Figure 5F, Figure S5), and the structure of PTH1R bound to P2 was also close to that of its parent, PTHrP(1–36) (Figure S3B). Thus, our structural comparison suggests that cryo-EM analysis of peptide-PTH1R-Gαs complexes may not capture functionally important differences in agonist-receptor interactions.

Overall, we hypothesize that the α→β substitutions in SPT lead to small improvements in residue packing that collectively stabilize the agonist-receptor interaction. This hypothesis seems consistent with the greater buried surface area between peptide and receptor for SPT compared to PTH(1–34) or PTHrP(1–36) (∼2030 Å^2^ vs 1990 Å^2^ or 1930 Å^2^, respectively). However, we acknowledge that buried surface area measurements are not directly correlated with complex stability and that the differences in buried surface area among these three structures are small.

## Conclusion

SPT, a peptide containing five β-amino acid residues that is derived from PTHrP(1–36), was recently shown to cause a substantially prolonged calcemic effect *in vivo* relative to PTH(1–34),^23^ and the studies described here were designed to explore the mechanistic origins of this prolonged signaling. Results presented in this study confirm that SPT potently induces disappearance of the PTH1R from the cell surface, and we show that the pattern of intracellular receptor trafficking caused by SPT differs from that caused by PTH(1–34) or ABL, both of which are approved for treatment of osteoporosis. The functional profile of SPT is reminiscent of the profile of LA-PTH, a PTH/PTHrP hybrid peptide that shows prolonged pharmacodynamic effects in vivo and prolonged cAMP signaling and internalization in cells. LA-PTH is a clinical candidate, as eneboparatide, for the treatment of hypoparathyroidism.^12,24,36,68^ Our results with SPT support the conclusion that engineered PTH1R agonists can display prolonged signaling by altering intracellular receptor trafficking. This mechanism of extending duration of action is distinct from the use of hydrophobic appendages to induce binding to serum albumin, which is employed in widely used GLP-1R agonists for treatment of type 2 diabetes and obesity.^6,29^

An initial qualitative survey of receptor location within cells after agonist stimulation was conducted via fluorescence microscopy. These experiments employed engineered versions of the receptor and a protein that marks early, late, or recycling endosomes (Rab5, Rab7, or Rab11, respectively),^39^ with the receptor fused to eGFP and the Rab protein fused to mCherry. Similar extents of PTH1R translocation to early endosomes were observed after cell treatment with PTH(1–34), ABL, or SPT, but no evidence of the receptor in late endosomes was detected for any peptide. There was a clear difference among the peptides in terms of recycling endosomes, which appeared to contain the PTH1R after cellular stimulation with PTH(1–34) or ABL but not after stimulation with SPT.

The qualitative endosomal colocalization patterns derived from fluorescence microscopy were complemented by quantitative data from a series of BRET assays in which the donor (a luciferase) was fused to the PTH1R, and the acceptor (Venus) was fused to one of the Rab proteins. These assays were consistent with the microscopy in showing comparable colocalization of the PTH1R to early endosomes after stimulation with each of the three peptide agonists. However, BRET assay results were not consistent with microscopy in terms of late endosomes, which target contents to lysosomes,^37^ because stimulation with all three peptides led to detection of the receptor in this compartment in the BRET assay but not via microscopy. This difference might reflect divergent influences of the distinct fluorescent tags employed in the two sets of experiments, or the different peptide concentrations used to saturate receptors (0.1 vs 1.0 μM), or perhaps microscopy is less sensitive than the BRET assays.

BRET assay results for recycling endosomes were consistent with microscopy in showing that both PTH(1–34) and ABL are much more effective at directing the PTH1R to this compartment relative to SPT. The trend in PTH1R sorting to Rab11-marked endosomes revealed by the BRET assays was correlated with results of a recycling assay, which showed that the PTH1R reappears at the cell surface very rapidly after cellular stimulation with ABL, more slowly after stimulation with PTH(1–34), and very slowly or not at all after simulation with SPT.

Internalization of the PTH1R following agonist engagement is mediated by recruitment of β-arrestin to the intracellular surface of the receptor.^37^ Continued production of cAMP after internalization depends on maintaining receptor-β-arrestin engagement, and cAMP production is terminated when β-arrestin is displaced by the retromer complex, which ultimately enables receptor recycling to the cell surface.^28^ However, β-arrestins can also terminate cAMP signaling by blocking the receptor from engaging Gαs, depending on the specific conformation that the β-arrestin adopts on the receptor.^57^ These features of PTH1R trafficking motivated us to generate assays to detect β-arrestin recruitment to the receptor at specific sites within the cell.

At the plasma membrane, no difference in stimulation of β-arrestin-2 recruitment to the PTH1R was observed among PTH(1–34), ABL and SPT. For β-arrestin-1, a small enhancement was observed for SPT relative to the other two peptides, which were indistinguishable from one another. In contrast, substantial differences were observed for recruitment of β-arrestin-1 or β-arrestin-2 to the PTH1R in endosomes. In both cases, cellular stimulation with SPT caused the highest level of β-arrestin recruitment to the receptor in endosomes, and stimulation with ABL caused the lowest level of β-arrestin recruitment in endosomes. The level of endosomal β-arrestin recruitment was inversely correlated with extent of receptor localization in recycling endosomes and with the rate at which the receptor reappeared at the cell surface. These observations are consistent with the hypothesis that enhanced receptor-β-arrestin engagement detected with the endosome-specific BRET assay delays the sorting of early endosomes into recycling endosomes. We propose that enhanced β-arrestin engagement hinders displacement of β-arrestin from the receptor by the retromer complex to terminate cAMP production. We further propose that the altered intracellular trafficking induced by SPT relative to PTH(1–34) underlies the prolonged signaling observed *in vivo* for SPT relative to PTH(1–34).

How can the identity of the peptide that initially activates the PTH1R at the cell surface influence the sorting of early endosomes containing the receptor into recycling endosomes? This correlation presumably depends on persistence of peptide-receptor complexes after internalization, at least for PTH(1–34) and SPT. Previous studies provided direct evidence for persistence of complexes between PTH(1–34) and the PTH1R within endosomes;^21,27^ these studies suggested that PTHrP(1–36) dissociates from the receptor upon internalization. Since ABL is a derivative of PTHrP(1–34), it is possible that this peptide, too, dissociates from the receptor after internalization. Transfer of information through a GPCR is thought to depend on distinct receptor conformations or conformational ensembles induced by agonist binding. Agonists can cause different outcomes by favoring different receptor conformations; this general mechanism is thought to underlie signal bias.^69^ We propose that the altered intracellular receptor trafficking induced by SPT relative to PTH(1–34) arises from a difference in agonist-induced receptor conformations, as suggested indirectly by results of the endosome-specific β-arrestin recruitment assays. We did not observe substantial differences between our cryo-EM structure of SPT bound to the PTH1R and a reported structure containing PTH(1–34).^62^ GPCRs in complex with G proteins that are nucleotide-free, as was the case for these PTH1R structures, can be limited in their ability to explain functional outcomes. These limitations have motivated recent development of methods to uncover additional states using cryo-EM.^70,71^ Future application of these methods to PTH1R complexes might reveal latent conformational variation underlying the divergent signaling of SPT vs PTH(1–34).

Several studies have documented peptide agonists of class B1 GPCRs that can induce cAMP production but are defective in terms of causing receptor internalization. Jones et al., for example, reported that the His1→Phe analogue of exendin-4 displays diminished β-arrestin recruitment relative to cAMP production (biased agonism) and diminished internalization at the GLP-1R.^30^ This analogue induces greater long-term insulin release and appears to cause fewer unfavorable side effects relative exendin-4. Wu et al. described other exendin-4 analogues with diminished β-arrestin recruitment and diminished internalization of the GLP-1R.^72^ Novikoff et al. showed that two dual GLP-1R/GIPR agonists display diminished β-arrestin recruitment at the GLP-1R relative to GLP-1 and at the GIPR relative to GIP, and diminished internalization of both receptors.^43^ White et al. reported that the d-Leu7 diastereomer of PTH(1–34) displayed diminished β-arrestin recruitment and diminished internalization of the PTH1R.^12^ Rodriguez et al. recently described a dual GLP-1R/GIPR agonist that does not recruit β-arrestin to either receptor or induce receptor internalization; this dual agonist is superior to unbiased agonists in terms of controlling body weight in mice.^73^

The behavior of SPT is fundamentally different from these prior examples that are biased away from β-arrestin recruitment and defective in terms of receptor internalization because SPT is comparable to PTH(1–34) in terms of global β-arrestin recruitment to the PTH1R and in terms of receptor internalization. Nevertheless, SPT displays a substantially prolonged duration of action relative to PTH(1–34) *in vivo*. The results reported here suggest that the unusual pharmacodynamic profile of SPT is related to the ability of this agonist to alter the intracellular trafficking pattern of the PTH1R relative to PTH(1–34); SPT hinders the sorting of receptor-containing early endosomes into recycling endosomes, apparently because of enhanced engagement of the SPT-bound receptor with β-arrestins relative to PTH(1–34)-bound receptor (Figure 6 A,B). The β-arrestin engagement effect is specific to endosomes relative to the plasma membrane, as revealed by new types of targeted β-arrestin recruitment assays.

**Figure 6:**
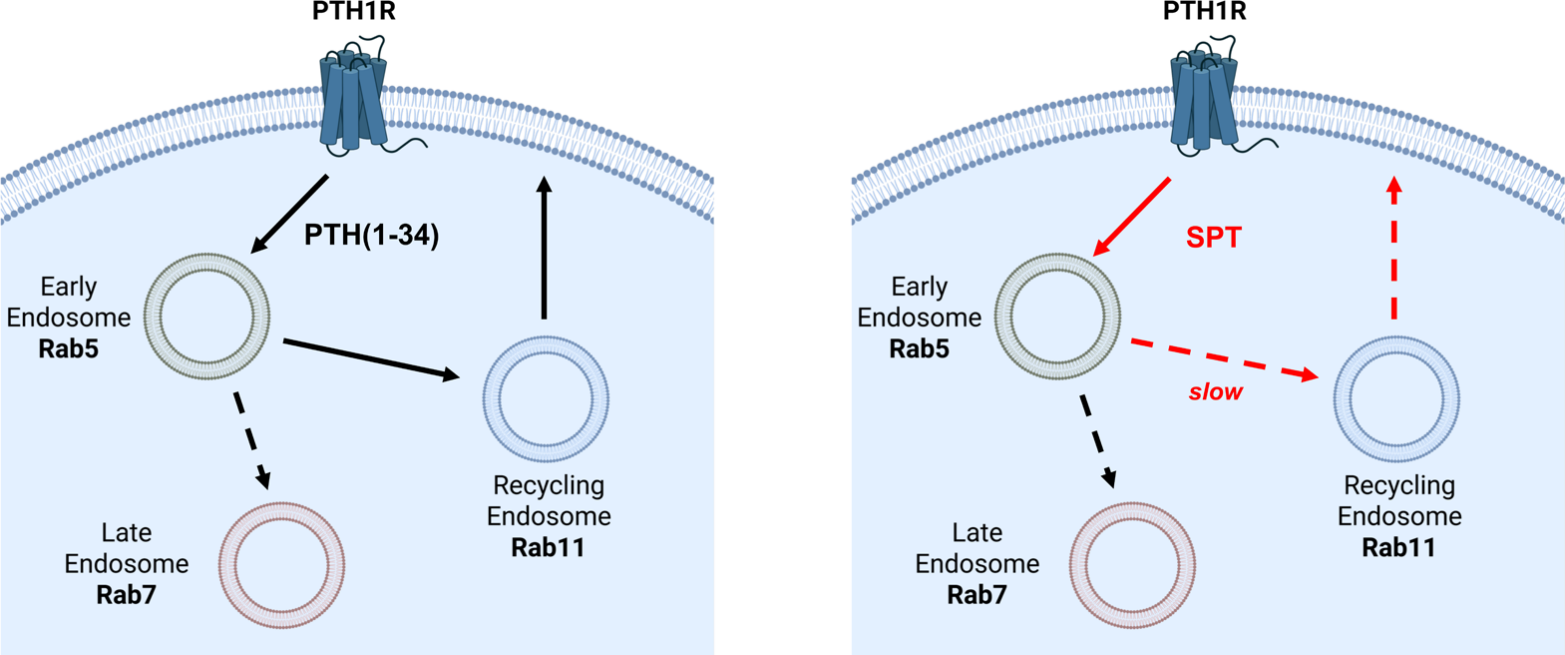
(A) Graphical summary of PTH1R trafficking that results from stimulation with PTH(1–34). Internalization is followed by rapid return of many receptors to the plasma membrane. (B) Graphical summary of our hypothesis to explain PTH1R trafficking that results from stimulation with SPT; sorting of early endosomes into recycling endosomes is hindered by receptor engagement with the α/β-peptide agonist. The cartoons were generated using Biorender.

Reports of agonist-dependent alteration of GPCR intracellular trafficking are rare,^38,43^ but such effects might prove to be widespread in this large receptor class. The findings reported here for SPT raise the possibility that enhanced β-arrestin recruitment to endosomal receptors may be a general path to class B1 GPCR agonists with prolonged duration of action. This strategy for extending activity *in vivo* is distinct from the use of hydrophobic appendages in agonists such as semaglutide.^6,29^

## Methods

### Peptide Synthesis

Peptides were prepared by microwave-assisted solid-phase peptide synthesis using either a CEM Mars microwave reactor (manual) or an automated CEM Liberty Blue reactor. Low-loading Rink Amide resin (CEM) was used as the solid support. For peptide coupling steps, 5 equiv of Fmoc-amino acid, 10 equiv of diisopropylcarbodiimide, and 5 equiv of Oxyma with respect to the resin loading were used. NMP or DMF solutions of these compounds were added to the reaction vessel. The resulting slurry was heated to 90 °C and stirred for 2 min, except for histidine, which was heated to 50 °C for 10–30 min. After the heating, the resin was washed with DMF thrice, and Fmoc was removed by adding 20% piperidine solution in DMF with 0.1% Oxyma, and heating to 80 °C for 2 min. For orthogonal removal of the alloc protecting group on a lysine side chain, to install a fluorophore, the resin was washed with methylene chloride thrice, and 10 equiv of phenylsilane and 1 equiv of tetrakis­(triphenylphosphine)­palladium(0) in ethylene dichloride were added to the reaction vessel. The resulting slurry was heated to 35 °C for 10 min. This process was repeated twice. The resin was washed with methylene chloride thrice and DMF thrice. To add the fluorophore, 4 equiv. 6-carboxytetramethylrhodamine, 4 equiv of diisopropylcarbodiimide, and 4 equiv of Oxyma in biotech-grade DMF were added, and the resulting mixture was heated to 75 °C for 10 min. At the end of the solid-phase synthesis, the peptide was cleaved and deprotected in a solution containing 90% TFA, 5% thioanisole, 3% 3,6-dioxa-1,8-octanedithiol (DODT) and 2% anisole by incubating at room temperature for 4 h. The resulting solution was filtered from the resin, and product was precipitated by addition of 10-fold excess (by volume) of diethyl ether that had been cooled to −20 °C. Collected precipitate was washed twice with diethyl ether, dried, and dissolved in DMSO for further purification with a preparative reverse-phase HPLC system (Waters) using a C-18 column (12 mL/min flow rate, with gradients of H_2_O + 0.1% TFA as solvent A and MeCN + 0.1% TFA as solvent B). The collected fractions were analyzed based on mass using MALDI-TOF-MS (Bruker UltraFlex) with CHCA as matrix. Isolated peptide was assessed for purity using a UPLC system (Waters) with a C-18 column (0.3 mL/min flow rate, with gradients of H_2_O + 0.1% TFA as solvent A and MeCN + 0.1% TFA as solvent B), detection at 220 nm. The concentration of peptide in stock solutions was determined using a NanoDrop One UV/vis system (Thermo Scientific), based on absorbance at 205 nm. The extinction coefficients of the peptides were calculated using Protein Parameter Calculator (http://nickanthis.com/tools/a205.html)

### Cell Culture

Cells were grown in Dulbecco’s Modified Eagle Medium (with d-glucose but lacking l-glutamine and sodium pyruvate) supplemented with 10% fetal bovine serum (Corning, Cat# 45000–734). Cells were incubated at 37 °C with 5% CO_2_. Dulbecco’s Phosphate Buffered Saline (DPBS; Gibco, Cat#14190235) and trypsin-EDTA (Corning, Cat# 45001–082) at 0.05% were used to detach cells for passaging and transfer to assay plates.

### Transfection

HEK293H cells were seeded onto a 100 mm dish (Fisher, Cat# FB012924) and grown to 80–90% confluency as observed under a microscope. On the day of transfection, 10 μg of plasmid was mixed with 30 μL of FugeneHD (Promega, Cat# PAE2311) in Opti-MEM (Gibco, Cat# 31985062) to bring the final volume to 1 mL. The resulting mixture was gently mixed by flicking the Eppendorf tube, which was set to incubate at room temperature for 10 min. The medium from the 100 mm dish was aspirated, and the cells were washed with DPBS without calcium or magnesium. To the cells was added 9 mL of DMEM with 10% FBS, and the transfection solution was added gently over the cells. The cells were left to incubate at 37 °C for 24–36 h.

### cAMP Glosensor Measurements in UMR106 Cells

UMR106 cells were grown in continuous culture in DMEM (Sigma) + 10% FBS at 37 °C and 5% CO_2_ in a humidified atmosphere. For live-cell cAMP measurements, cells were transfected in suspension with a plasmid containing the coding sequence for the cAMP sensor Glosensor 22F (Promega) using PEI and seeded directly into white flat bottomed 96 well plates and incubated for approximately 48 h. On the day of the experiment, cells were washed 2x in assay buffer (Hanks’ balanced salt solution +1% ovalbumin) before adding assay buffer with 500 μM D-luciferin and left protected from light at room temperature for 1 h. Luminescence was read on a BMG Pherastar in plate mode with a 1s interval time and 120s cycle time. After a baseline reading, ligands serially diluted in assay buffer were added using a multichannel pipet. Prewash concentration–response data were generated by calculating the area under the curve for five measurement cycles before washout. Each biological replicate was normalized with the response of PTH(1–34) at 1 μM concentration representing 100%. Three parameter curves were fitted with shared bottom value.

After five read cycles following ligand addition, the plates were washed 2x in assay buffer and resupplied with assay buffer containing 500 μM D-luciferin for the remainder of the experiment. To minimize the effect of washing, all data were normalized first to the average baseline luminescence (each well independently) and then normalized to the average signal from vehicle-treated well. All data normalization and processing were performed in either GraphPad Prism or Microsoft Excel.

### Competition Binding Assay Using BRET

HEK293H cells stably expressing the nLuc-PTH1R were generated using zeocin resistance as the selective marker. The cells were grown in DMEM supplemented with 10% FBS. Before the day of the experiment, the cells were seeded onto a white opaque 96-well assay plate at 5,000–10,000 cells per well. The cells were incubated at 37 °C for 24 h. On the day of the experiment, the medium was aspirated, and 70 μL of DPBS supplemented with 0.02% NaN_3_, 1 mM CaCl_2_ and 0.5 mM MgCl_2_ was added. The cells were incubated at room temperature for at least 30 min before the experiment. The medium was aspirated, and 80 μL of fresh DPBS with the same composition was added. Ten μL of the labeled tracer peptide solution (PTH(1–34)-K35^TMR^; ref 33) was prepared in the same solution and added to the cells to bring the final tracer concentration to 30 nM. Ten μL of the unlabeled peptides at varying concentrations were added to bring the final volume to 100 μL, and the cells were incubated for 1 h. For the experiment, 10 μL of 11X h-coelenterazine solution was added to the well, and the luminescence from 460 and 590 nm was measured over 30 min at 1 min intervals. The ratio of the luminescence from the 590 nm channel over that from the 460 nm channel with a 1,000x multiplier at the 10 min time point was used as the mBRET value for generating plots. Curves were generated with Graphpad Prism 9 software using a log­(concentration) vs response nonlinear regression model.

### Confocal Fluorescent Microscopy

HEK293FT cells were cultured at 37 °C with 5% CO_2_ and elevated humidity. One day before the experiment, the cells were transfected with 1 μg of eGFP-PTH1R and 1 μg of mCherry-Rab­(5/7/11) proteins in 100 uL Opti-MEM supplemented with 6 μL of FugeneHD (Promega) on a 35 mm confocal cell culture-treated dish (CellTreat). The transfected cells were incubated at 37 °C with 5% CO_2_ for 24 h. On the day of the experiment, the medium was changed back to complete Fluorobrite DMEM (Gibco) supplemented with 10% FBS (Corning), and the cells were incubated for 30 min. The cells were treated with ligand at 100 nM final concentration for 30 min at 37 °C. The ligand-containing medium was washed out and replaced with Fluorobrite DMEM supplemented with 10% formalin solution for 15 min. The cells were observed under Nikon A1Rs confocal microscope at 60x oil-immersion objective lens with 588 nm (eGFP) and 561 nm (mCherry) laser wavelengths. The acquired fluorescent micrographs were processed with ImageJ software.

### BRET Measurement of PTH1R Trafficking

HEK293H cells were seeded on a 10 cm dish and grown until 70–80% confluence. The cells were transfected with 0.4 μg of PTH1R-nLuc and 4.5 μg of Venus-Rab­(5/7/11) in 1 mL of Opti-MEM (Gibco) supplemented with 15 μL of FugeneHD (Promega). The cells were incubated for 24 h and then harvested using trypsin (0.05%). One-third of the cells from the dish were evenly seeded on a white 96-well plate in 100 μL/well and incubated for 24 h. On the day of the experiment, the medium was aspirated, and the cells were washed once with 100 μL of room-temperature Hank’s balanced salt solution (HBSS). 90 μL of HBSS containing furimazine (5 μM) was added and incubated at room temperature in the dark for 5 min. Ten μL of agonist or vehicle was added to bring the final agonist concentration to 1 μM, and the luminescence at two wavelengths (480 nm, 530 nm) were monitored for 30 min. The ratio of the two values (lum_530_/lum_480_) was multiplied by a factor of 1,000 to yield mBRET values at each time point. The resulting BRET response from the vehicle-treated well was subtracted from the agonist-treated wells, and the difference values were plotted on a time course graph.

### PTH1R β-Arrestin Recruitment

HEK293H cells grown on a 10 cm dish were transfected with 0.4 μg of PTH1R-nLuc and 4.6 μg of HaloTag-β-arrestin1 or HaloTag-β-arrestin2 in 1 mL of Opti-MEM supplemented with 15 μL FugeneHD. The cells were incubated for 24 h and harvested using trypsin. A third of the cells from the dish were reconstituted in complete DMEM containing TMR-HaloTag substrate (5 μM) and evenly seeded on a white 96-well plate, which was then incubated for 24 h. On the day of the experiment, the medium was aspirated, and the cells were washed once with 100 μL HBSS. 90 μL of HBSS containing furimazine (5 μM) was added, followed by the agonists at varying concentrations. The luminescence values at two wavelengths (450 nm, 610 nm) was monitored for 30 min, and the ratio of the two luminescence values (lum_610_/lum_450_) was plotted against the concentration of the agonist using Graphpad Prism 9 software and with a log­(concentration) vs response nonlinear regression model.

### Detection of PTH1R:β-Arrestin Complexes at the Endosomes or Plasma Membrane

HEK293H cells grown on a 10 cm dish were transfected with 0.1 μg of PTH1R-smBiT, 0.1 μg of LgBit-Endofin (endosome) or LgBiT-CaaX (plasma membrane), and 1.8 μg of HaloTag-β-arrestin1 or HaloTag-β-arrestin2 in 100 μL of Opti-MEM supplemented with 6 μL FugeneHD. The cells were incubated for 24 h and harvested using trypsin. A third of the cells from the dish were reconstituted in complete DMEM containing NanoBRET618-HaloTag substrate (5 μM) and evenly seeded on a white 96-well plate, which was then incubated for 24 h. On the day of the experiment, the medium was aspirated, and the cells were washed once with 100 μL HBSS. 90 μL of HBSS was added followed by 10 μL of the agonist or vehicle to bring the final agonist concentration to 1 μM. The cells were allowed to equilibrate in the presence of each agonist by incubating at 37 °C for 30 min, and 1 μL of furimazine was added to bring the final concentration of furimazine to 5 μM. The resulting BRET response was monitored for 30 min, and the AUC values measured over 30 min were plotted using Graphpad Prism 9 software.

### HiBiT-PTH1R Recycling

HEK293H cells were transfected with 0.2 μg of HiBit-PTH1R and 1.8 μg of carrier DNA in 1 mL of Opti-MEM supplemented with 6 μL of FugeneHD on a 10 cm dish. The cells were incubated at 37 °C for 24 h and harvested using trypsin. Half of the cells from the dish were evenly seeded on a white 96-well plate and incubated for 24 h. On the day of the experiment, the medium was aspirated and 100 μL of CO_2_-independent medium containing cycloheximide (100 μM) was added to each well. One μL of the agonist or vehicle was added to bring the final agonist concentration to 100 nM, and the cells were incubated at 37 °C for 30 min. The medium was aspirated, and 100 μL of fresh media containing furimazine (5 μM), cycloheximide, PTH(3–34) (100 nM), and LgBiT (1:1,000) protein was added. The resulting luminescence was monitored for 1 h. To measure the effect of the phosphatase inhibitor, okadaic acid (1 μM) was added during the incubation with the agonist, and the luminescence was monitored for 30 min. The resulting luminescence was normalized against the vehicle-treated wells across the time frame of the measurement.

### HiBiT-PTH1R Internalization

HEK293H cells were transfected with 0.2 μg of HiBit-PTH1R and 1.8 μg of carrier DNA in 1 mL of Opti-MEM supplemented with 6 μL of FugeneHD on a 10 cm dish. The cells were incubated at 37 °C for 24 h and harvested using trypsin. Half of the cells from the dish were evenly seeded on a white 96-well plate and incubated for 24 h. On the day of the experiment, the medium was aspirated, and 90 μL of CO_2_-independent media was added to each well, followed by 10 μL of the agonists at varying concentrations. The cells were incubated at 37 °C for 30 min. Ten μL of medium containing LgBiT protein (1:50) and 11X of furimazine substrate (55 μM) was added, and the resulting luminescence was monitored for 30 min. The luminescence values at the 10 min mark were used to plot the concentration–response curves, and a fit was generated with Graphpad Prism 9 software using a nonlinear regression log­(concentration) vs response model.

### Statistics

Graphpad Prism 9 software was used for all quantitative analysis. Multiple comparisons were analyzed using one-way ANOVA followed by Dunnett’s multiple comparisons.

### DNA Constructs and Molecular Biology

mCherry-Rab5, mCherry-Rab7, and mCherry-Rab11 were generous gifts from the Voeltz lab, acquired through Addgene. Venus-Rab5, PTH1R-nLuc, PTH1R-smBiT HiBiT-PTH1R, LgBiT-CAAX, and Endofin-LgBiT were synthesized by Twistbio in the pTwist plasmid backbone. Venus-Rab7 and Venus-Rab11 were constructed by restriction cloning the donor Rab7 and Rab11 constructs into the Venus-Rab5 acceptor plasmid. HaloTag-β-arrestin1 and HaloTag-β-arrestin2 were synthesized by Gibson assembly into pHTN plasmid backbone (Promega).

### PTH1R/Gs Complex Formation

The SPT/PTH1R/Gs complex was prepared and concentrated to 5.25 mg/mL as previously described^62^ with minor alterations to the procedure. A *Trichoplusia ni* (High Five) cell pellet expressing PTH1R, DNGs, Gβ1 and Gγ2 from 0.5 L of culture was thawed and resuspended in 40 mL buffer (40 mL, 30 mM HEPES, 50 mM NaCl, 2 mM MgCl_2_, and 5 mM CaCl_2_, pH 7.4).^74^ To this mixture was added 5 μM agonist peptide, EDTA-free protease inhibitor (two tablets), nanobody 35 (∼600 μg),^75^ benzonase (2 μL) and apyrase (5 μL). The resulting mixture was stirred overnight at 4 °C. Complex solubilization, anti-FLAG affinity purification, and size-exclusion chromatography followed literature precedent.^62^ The PTH1R/Gs complex with peptide 2 was purified from a *Trichoplusia ni* cell pellet (from 1 L of culture) and concentrated to 5.7 mg/mL, as described above. The concentrated sample was flash frozen in liquid nitrogen and stored at −80 °C.

### Cryo-EM Sample Preparation

After size exclusion chromatography, fractions containing the SPT/PTH1R/Gs complex were pooled, concentrated to 5.25 mg/mL (100 kDa MWCO), and stored briefly (∼1 h) on ice before vitrification. Quantifoil grids (200 mesh, r1.2/1.3, Au-coated) were glow discharged (GloQube Plus, air chamber, 20 mA, 60 s, negative polarity). Sample (3 μL) was applied to the grids. Using a Vitrobot MkIV (Thermo Fisher, 4 °C, 96% humidity), the grids were blotted (6 s, blot force 17) and plunge frozen in liquid ethane. For PTH1R/Gs with peptide 2, the grid was prepared in the same manner, except the frozen sample was thawed and kept on ice before application.

### Cryo-EM Data Acquisition

The grids prepared with SPT/PTH1R/Gs samples were clipped and loaded into a Thermo Fisher Scientific Glacios (200 kV, Falcon 4). The instrument was operated with an indicated magnification of 120,000 in nanoprobe mode with a pixel size of 0.878 Å. An objective aperture (100 μm) and C2 aperture (50 μm) were used. Automated data collection was performed using aberration free image shift (AFIS) as implemented in EPU 2 software (Thermo Fisher Scientific). Data was saved as electron-event representation (EER) files. The total dose applied was 49.93 e^–^/Å^2^ at a dose rate of 6.13 e^–^/px/s.For the grid prepared with PTH1R/Gs with peptide 2 (P2), data were acquired on a Thermo Fisher Scientific Glacios microscope (200 kV) equipped with a Falcon 4i detector. Data were collected at an indicated magnification of 120,000× (0.86 Å pixel size) in nanoprobe mode with a defocus range of – 0.8 to – 1.2 μm. A 100-μm objective aperture and a 50-μm C2 aperture were used. Automated data collection was carried out using aberration-free image shift (AFIS) as implemented in EPU 2 software (Thermo Fisher Scientific). Multi frame (57 frames) images were saved as compressed tiff files. The total dose was 50 e–/Å^2^ applied over 6.59 s.

### Cryo-EM Data Processing

EER files were preprocessed using the EPU_Group_AFIS.py script (https://github.com/DustinMorado/EPU_group_AFIS) for import into RELION 3.1.2. MotionCor2 as implemented in RELION 3.1.2 ^76^ was used for patch motion correction.^77^ Contrast transfer function (CTF) estimation was performed with CTFFIND 4.1.14,^78^ and micrographs were selected by estimated maximum resolution. Particle picking was performed with crYOLO 1.7.6.^79^ Multiple rounds of 2D-classification and *ab initio* reconstruction were performed with cryoSPARC 4.1.2.^80^ Bayesian polishing was performed with RELION 3.1.2.^81^ Refinement with defocus and CTF refinement was performed with cryoSPARC 4.1.2. Details of the processing can be found in Figure S2. For processing of the PTH1R/Gs complex with P2 data set, compressed TIFF files were preprocessed using the EPU_Group_AFIS.py script (https://github.com/DustinMorado/EPU_group_AFIS) before import into RELION 5.0. MotionCor3 (https://github.com/czimaginginstitute/MotionCor3.git) was used for patch motion correction. Motion-corrected micrographs were imported into cryoSPARC 4.6.0, where CTF estimation was performed using patch CTF estimation. Map sharpening for display in figures was performed using DeepEMhancer.^82^ Details of data processing are provided in Figure S5.

### Atomic Modeling

The cryo-EM consensus map and the model of PTHrP bound to PTH1R (PDB: 8FLR) were loaded into ChimeraX 1.3.^83^ ISOLDE 1.3 ^84^ was used to perform flexible fitting. Ligands and link files were prepared using JLigand, as implemented in CCP4i version 7.1.017. Coot 0.9.6, as implemented in CCP4, was used for the manual refinement. Real-space refinement and validation were performed using Phenix 1.19.2.^85^ Buried area calculations were performed using ChimeraX 1.7 after the deletion of waters.

## Supplementary Material





## Notes

Video S1. 3D-variability analysis of B1 GPCR agonists (MP4)
Supporting figures for cell-based assays, fluorescent microscopy, protein preparation, cryoEM analysis, and peptide characterizations (PDF)
