# Supplementary material for: Altered Intracellular Trafficking as a Mechanism for Prolonged Duration of G Protein-Coupled Receptor Activation
Source: J Am Chem Soc. 2026 May 20;148(21):22045–61. doi: 10.1021/jacs.6c02192 (PMC13244458; doi:10.1021/jacs.6c02192)
Supplement: Supplementary file 2 [file ja6c02192_si_002.pdf]

Altered Intracellular Trafficking as a Mechanism for Prolonged Duration of G Protein-coupled Receptor Activation

Tae Wook Kim,[a] Elliot J. Gerrard,[b] Jeeeun Shin,[b,c] Thomas J. Gardella,[d] Denise Wootten,[b,c,*] Patrick M. Sexton,[b,c,*] Brian P. Cary[b,c,*] and Samuel H. Gellman[a,*]

Affiliations

[a] *Department of Chemistry, University of Wisconsin – Madison, Madison, Wisconsin 53706, United States.*

[b] *Drug Discovery Biology Theme, Monash Institute of Pharmaceutical Sciences, Monash University, Parkville 3052, VIC, Australia.*

[c] *ARC Centre for Cryo-Electron Microscopy of Membrane Proteins, Monash Institute of Pharmaceutical Sciences, Monash University, Parkville 3052, VIC, Australia.*

[d] *Endocrine Unit, Massachusetts General Hospital and Harvard Medical School, Boston, Massachusetts 02114, United States.*

** Corresponding Authors*

Email: patrick.sexton@monash.edu, denise.wootten@monash.edu, gellman@chem.wisc.edu or Brian.Cary@monash.edu

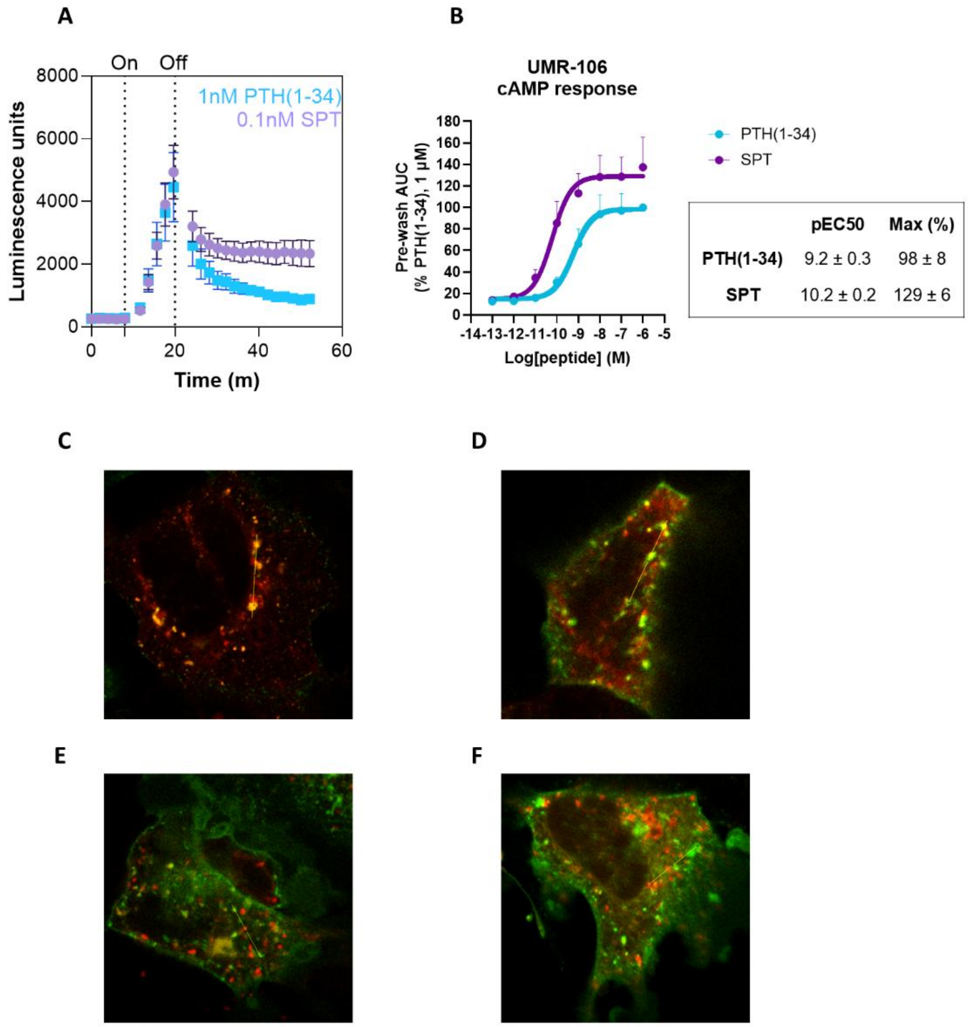

Figure S1. (A) cAMP washout assay using UMR-106 cells transiently transfected with a luminescent cAMP biosensor (Glosensor). Agonists near their EC_{50} concentrations (1 nM PTH or 0.1 nM SPT) were added to the cells at the timepoint indicated by "on", and a washout step was performed at the timepoint indicated by "off." the data represents mean ± standard error. n = 4, in duplicate. (B) Normalized concentration-response cAMP measurements, using transiently transfected Glosensor protein, in UMR106 cells. The inset table shows the negative log of the half-maximal effective dose (pEC50) and the maximal responses for the two peptides. N = 4 in duplicate. Error bars indicate SEM. Uncertainties for fitted values are symmetrical approximate standard errors. (C,D) Micrographs representing the location of PTH1R and Rab5-mCherry after stimulation by ABL or SPT. Cells expressing PTH1R-eGFP and Rab5-mCherry were stimulated by 100 nM ABL (C) or 100 nM SPT (D) for 30 minutes, fixed, then observed under a confocal microscope via 588 nm and 561 nm emission channels. (E,F) Micrographs representing the location of PTH1R and Rab7-mCherry after stimulation by ABL or SPT. Cells expressing PTH1R-eGFP and Rab7-mCherry were stimulated by 100 nM ABL (E) or 100 nM SPT (F) for 30 minutes, fixed, then observed under a confocal microscope via 588 nm and 561 nm emission channels.

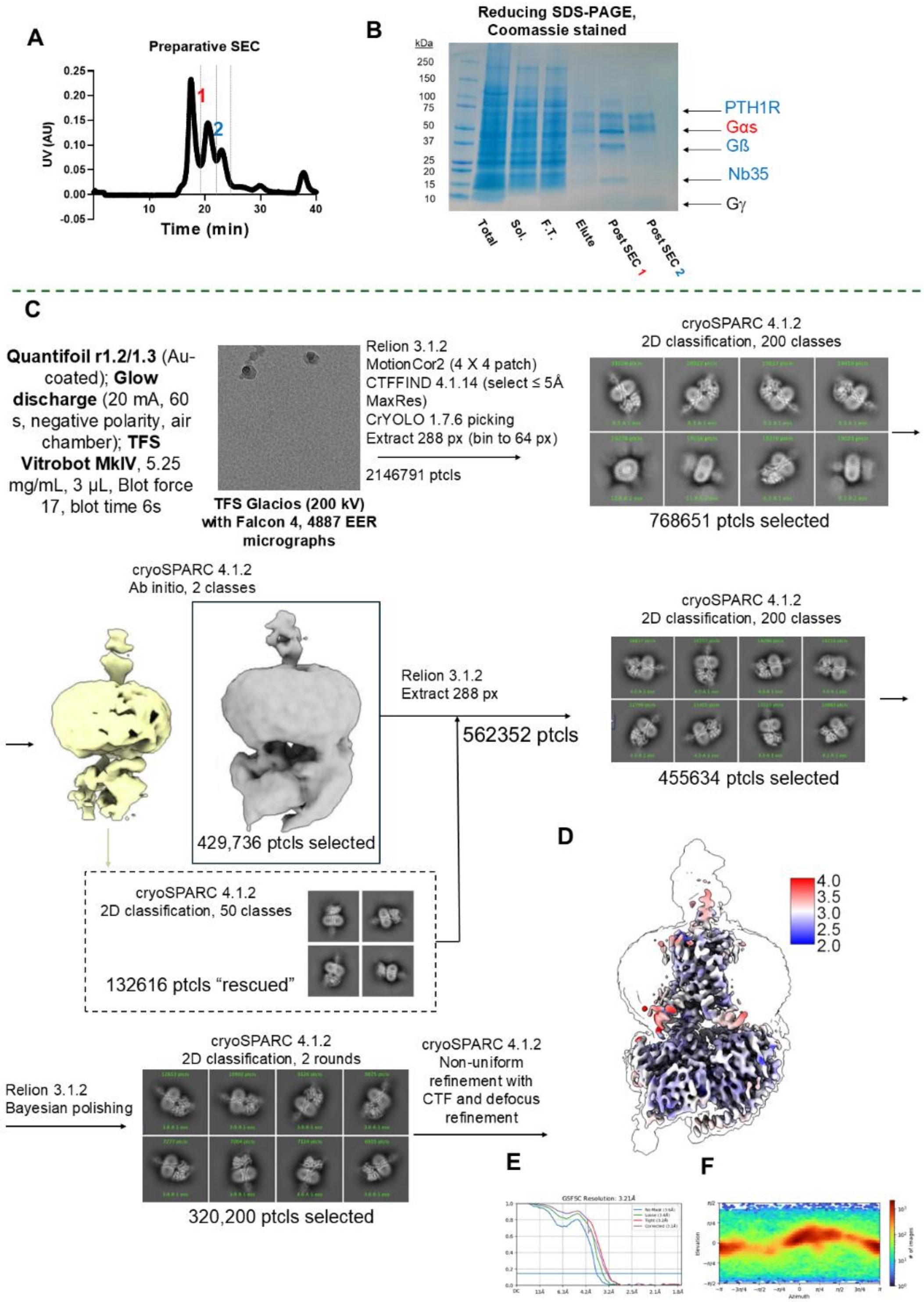

Figure S2. (A) Preparative size exclusion chromatography (SEC) trace for the sample containing peptide SPT and PTH1R. For the post-SEC samples labeled 1 and 2, fractions were collected and pooled approximately within the retention times indicated by the labels and dashed lines. (B) A reducing, denaturing gel containing samples from the protein preparation. The gel was stained by Coomassie staining.

“Sol.” Indicates the solubilized fraction, “elute” indicates the anti-FLAG affinity elution, and “post SEC” samples are after purification by size-exclusion. Post-SEC 1 was used for cryo-EM studies. (C) An overview of cryo-EM data acquisition and processing workflow. The field of view for the representative micrograph is approximately 360 nm x 360 nm. (D) A map of the consensus refinement with the surface colored by estimated local resolution. (E) The Fourier shell correlation (FSC) plot for the consensus refinement. (F) The particle distribution histogram for the consensus refinement.

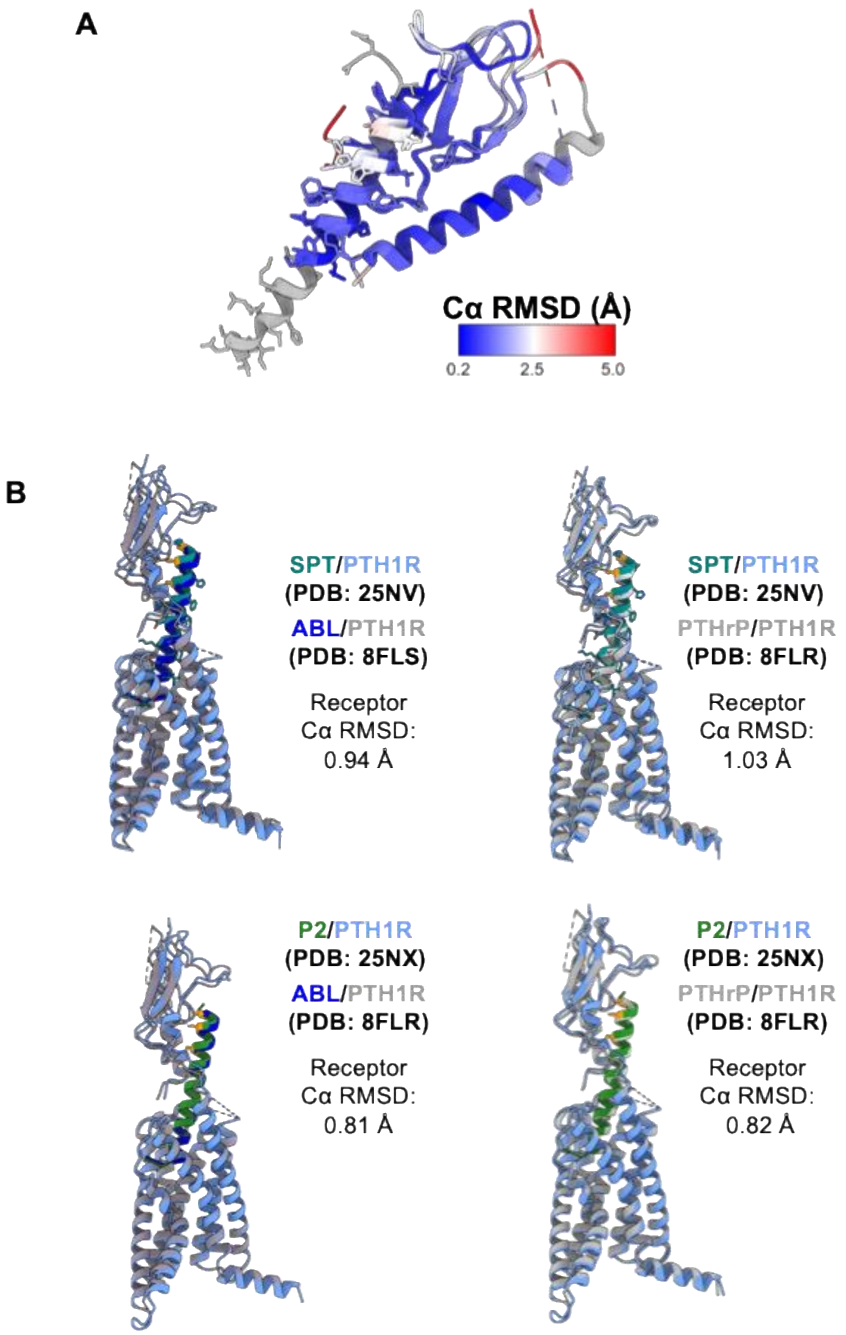

Figure S3: (A) Aligned structures of the cryo-EM structure of SPT/PTH1R (PDB: 25NV, ECD only) and the crystal structure of the PTH1R ECD bound to SPT (PDB: 7UZP). The backbones are colored by RMSD according to the key. Residues that are not included in both models are colored gray. (B) Additional structural alignments of the PTH1R TMD bound to either SPT (above) or P2 (below) with the PTH1R TMD bound to either ABL (left) or PTHrP (right).

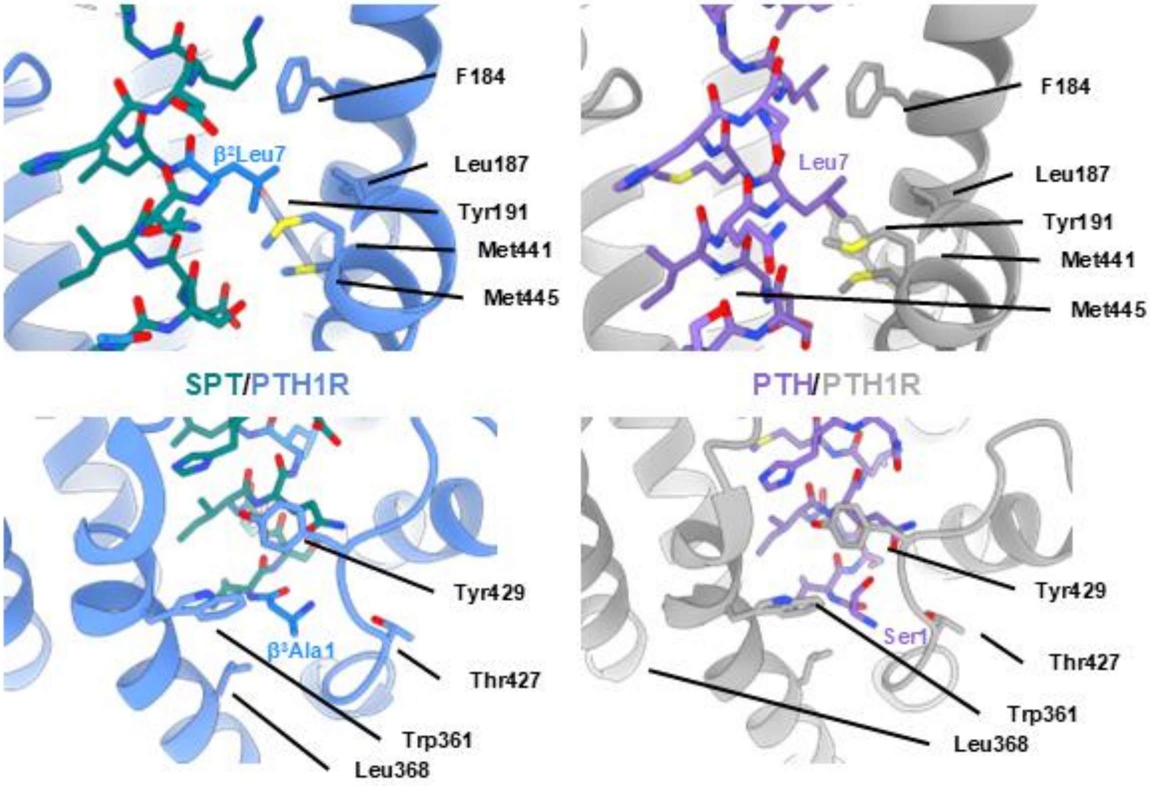

Figure S4. The binding pockets for peptide agonist residues 1 and 7 (bottom and top). SPT (25NV, left) contains β-amino acids at these two positions, and the structure containing PTH(1-34) (8FLQ, right) is shown for comparison. Select PTH1R residues in the vicinity of the peptide agonist residues are labeled and shown with stick formatting.

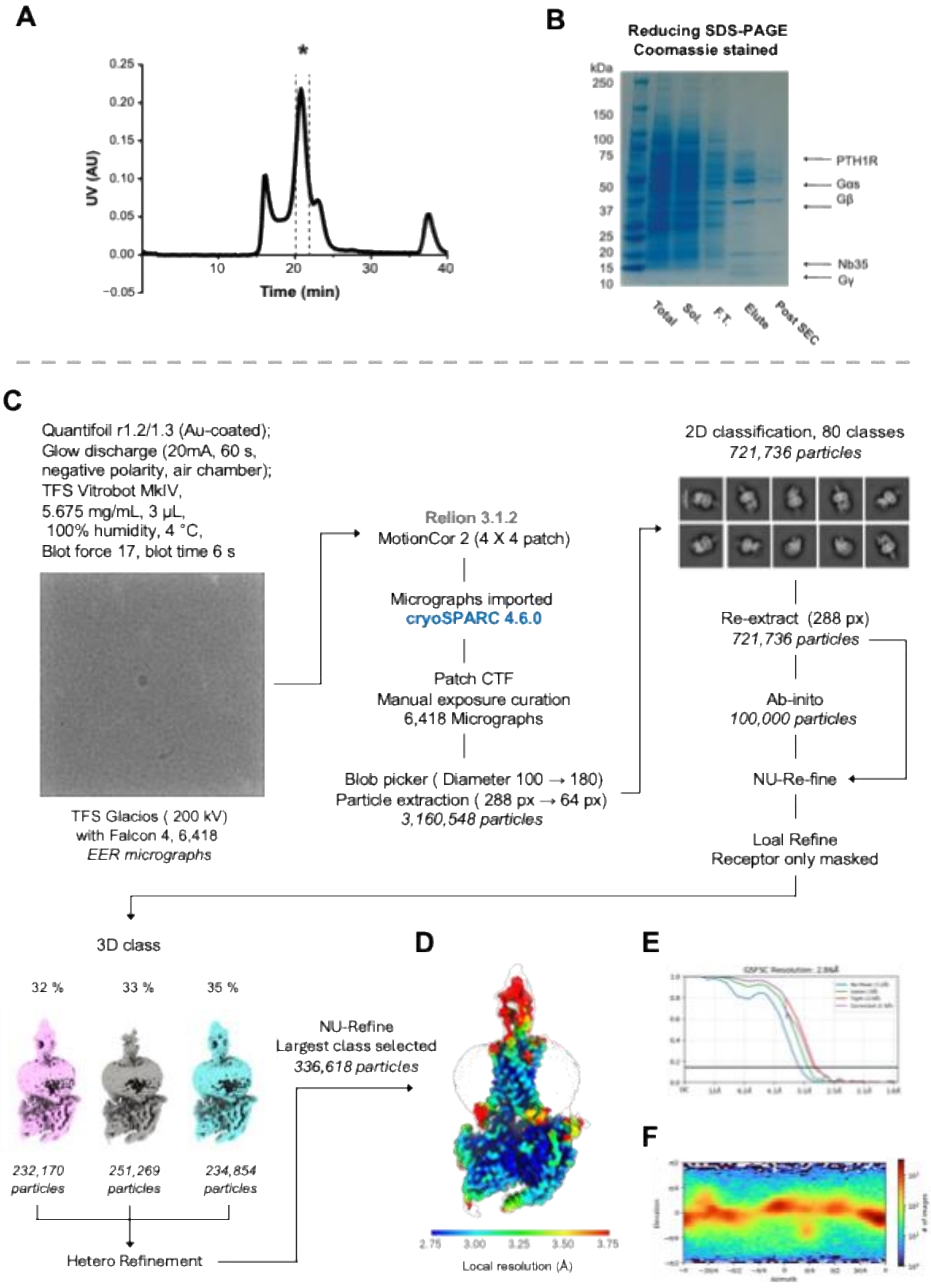
A
*
UV (AU)
0.25
0.20
0.15
0.10
0.05
0.00
−0.05
10
20
30
40
Time (min)
B
Reducing SDS-PAGE
Coomassie stained
kDa
250
150
100
75
50
37
25
20
15
10
PTH1R
Gαs
Gβ
Nb35
Gγ
Total
Sol.
F.T.
Elute
Post SEC
C
Quantifoil r1.2/1.3 (Au-coated);
Glow discharge (20mA, 60 s,
negative polarity, air chamber);
TFS Vitrobot MkIV,
5.675 mg/mL, 3 μL,
100% humidity, 4 °C,
Blot force 17, blot time 6 s
TFS Glacios (200 kV)
with Falcon 4, 6,418
EER micrographs
Relion 3.1.2
MotionCor 2 (4 X 4 patch)
Micrographs imported
cryoSPARC 4.6.0
Patch CTF
Manual exposure curation
6,418 Micrographs
Blob picker (Diameter 100 → 180)
Particle extraction (288 px → 64 px)
3,160,548 particles
2D classification, 80 classes
721,736 particles
Re-extract (288 px)
721,736 particles
Ab-inito
100,000 particles
NU-Re-fine
Loal Refine
Receptor only masked
3D class
32 %
33 %
35 %
232,170
particles
251,269
particles
234,854
particles
Hetero Refinement
NU-Refine
Largest class selected
336,618 particles
D
2.75
3.00
3.25
3.50
3.75
Local resolution (Å)
E
F

Figure S5. (A) Preparative size exclusion chromatography (SEC) trace for the sample containing peptide 2 (P2) and PTH1R. For the post-SEC sample, fractions were collected and pooled approximately within the retention times indicated by an asterisk. The sequence of peptide 2 is shown below the SEC trace, with X's indicating (S,S)-ACPC residues. (B) A reducing, denaturing gel containing samples from the protein preparation. The gel was stained by Coomassie staining. "Sol." indicates the solubilized fraction, "elute"indicates the anti-FLAG affinity elution, and "post SEC" samples are after purification by size-exclusion. The final pooled fraction from SEC gel-filtration was used for cryo-EM grid preparation. (C) An overview of cryo-EM data acquisition and processing workflow. The field of view for the representative micrograph is approximately 360 nm x 360 nm. (D) A map of the consensus refinement with the surface colored by estimated local resolution (Å). (E) The Fourier shell correlation (FSC) plot for the consensus refinement. (F) The particle distribution histogram for the consensus refinement.

**Imaging**	*SPT/PTH1RGs*	*P2/PTH1R/Gs*
PDB	25NV	25NX
EMDB	EMD-80237	EMD-80238
Magnification	120,000	120,000
Accelerating voltage	200 kV	200 kV
Electron exposure ($e^-/Å^2$)	49.93	50
Exposure time (s)	8.15	6.59
Movie frames	*n/a* (EER)	57
Pixel size (Å)	0.878	0.86
AFIS	Yes	Yes
Movies recorded	4887	6463
**Processing**		
Symmetry imposed	C1	C1
Initial particle images (no.)	2146791	3160548
Final particle images (no.)	320200	336618
Resolution (Å)	3.21	2.86
FSC threshold	0.143	0.143
**Refinement**		
Initial model used	*De novo*	*De novo*
B-factor	124.6	109.3
**Model composition**		
Chains	6	6
Non-Hydrogen Atoms	8775	9040
Protein residues	1139	1158
Waters	1	0
Ligands	5	3
**RMSDs**		
Bond length (Å)	0.006	0.006
Bond angles (°)	0.896	1.014
**Validation**		
MolProbity score	1.67	1.54
Clashscore	4.92	8.53
Rotamer outliers (%)	1.71	0.43
**Ramachandran plot**		
Favored (%)	96.48	97.61
Allowed (%)	3.34	2.39
Outliers (%)	0.18	0

Table S1. Cryo-EM data acquisition, processing and model parameters and statistics.

Peptide Characterization

PTH[1-34]-35K^{TMR} ; SVSEIQLMHN LGKHLNSMER VEWLRKKLQD VHNF-K^{TMR}-NH_2

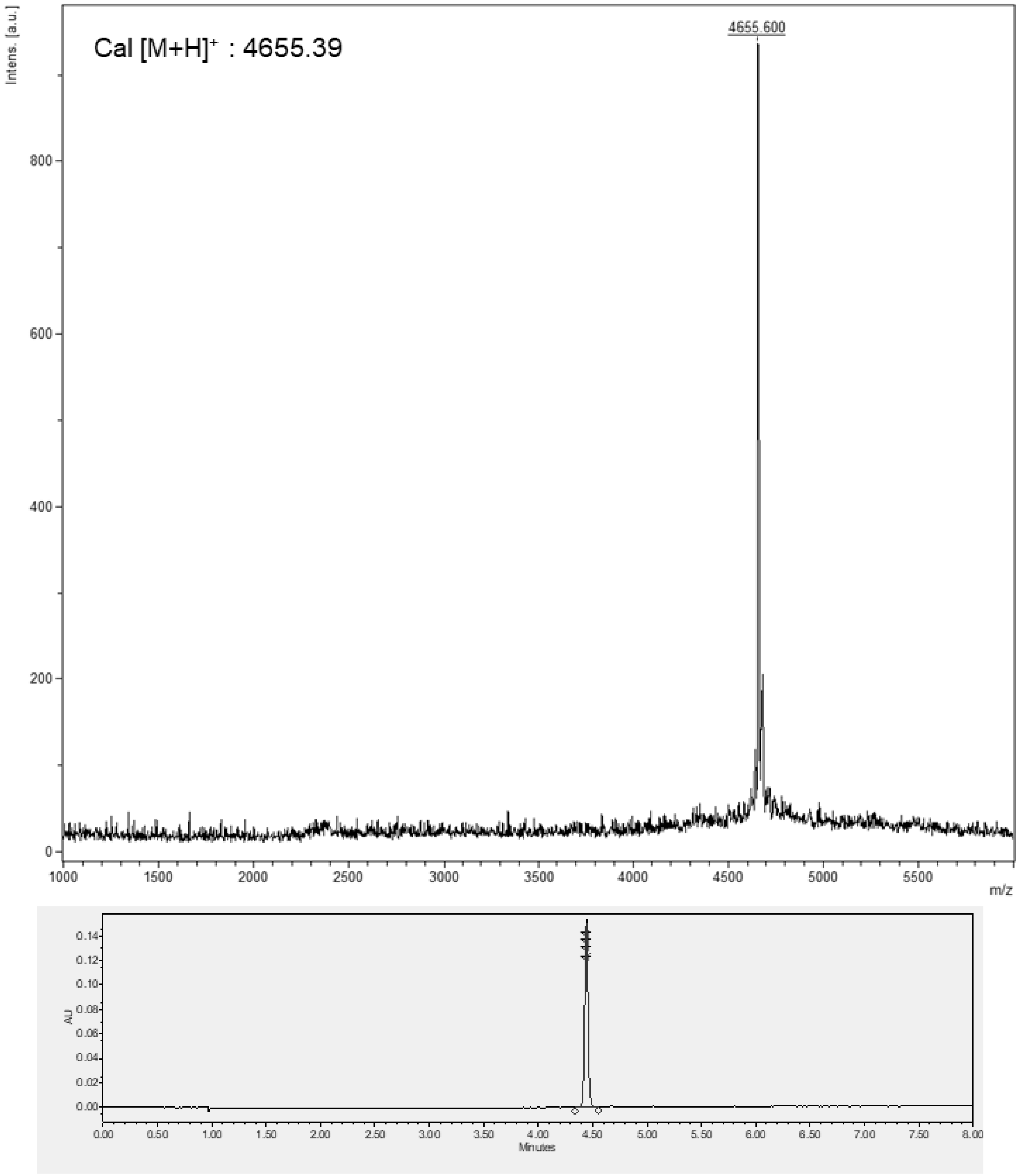

MALDI-TOF-MS (top) and UPLC (bottom) analysis of the PTH[1-34]-35K^{TMR} peptide. UPLC chromatogram at 220 nm was obtained with a ACQUITY UPLC BEH C18 column (2.1mm X 100mm) eluted with a linear gradient of 10-90% acetonitrile in water (0.1% TFA) applied over 8 min at a flow rate of 0.3 mL/min. Purity >95%.

PTH[1-34] ; SVSEIQLMHN LGKHLNSMER VEWLRKKLQD VHNF-NH_2

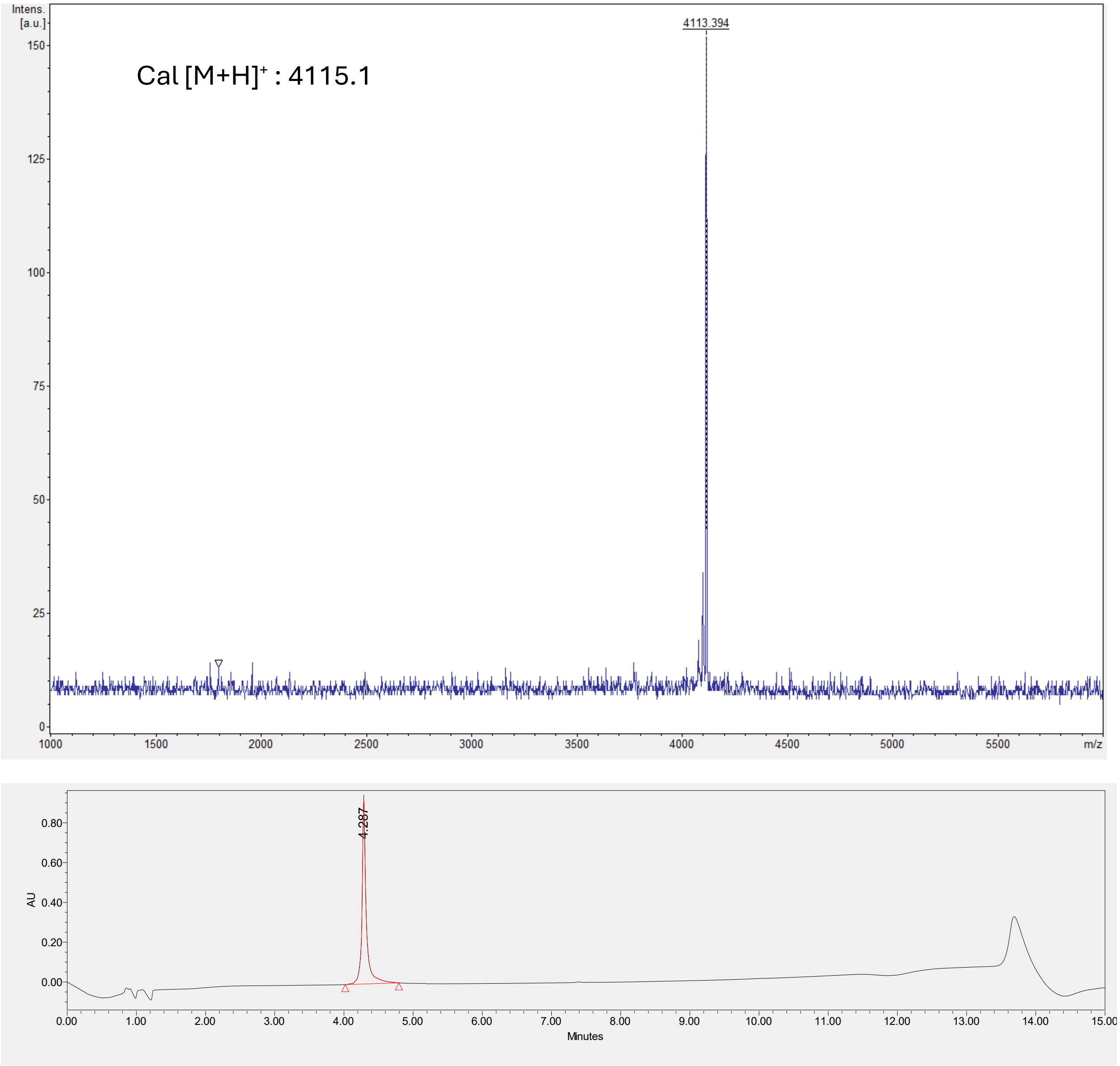

MALDI-TOF-MS (top) and UPLC (bottom) analysis of the PTH(1-34) peptide. UPLC chromatogram at 220 nm was obtained with a ACQUITY UPLC BEH C18 column (2.1mm X 100mm) eluted with a linear gradient of 10-90% acetonitrile in water (0.1% TFA) applied over 10 min at a flow rate of 0.3 mL/min. Purity >95%.

ABL ; AVSEHQLLHD KGKSIQDLRR RELLEKLLUK LHTA-NH_2

(U: Aib)

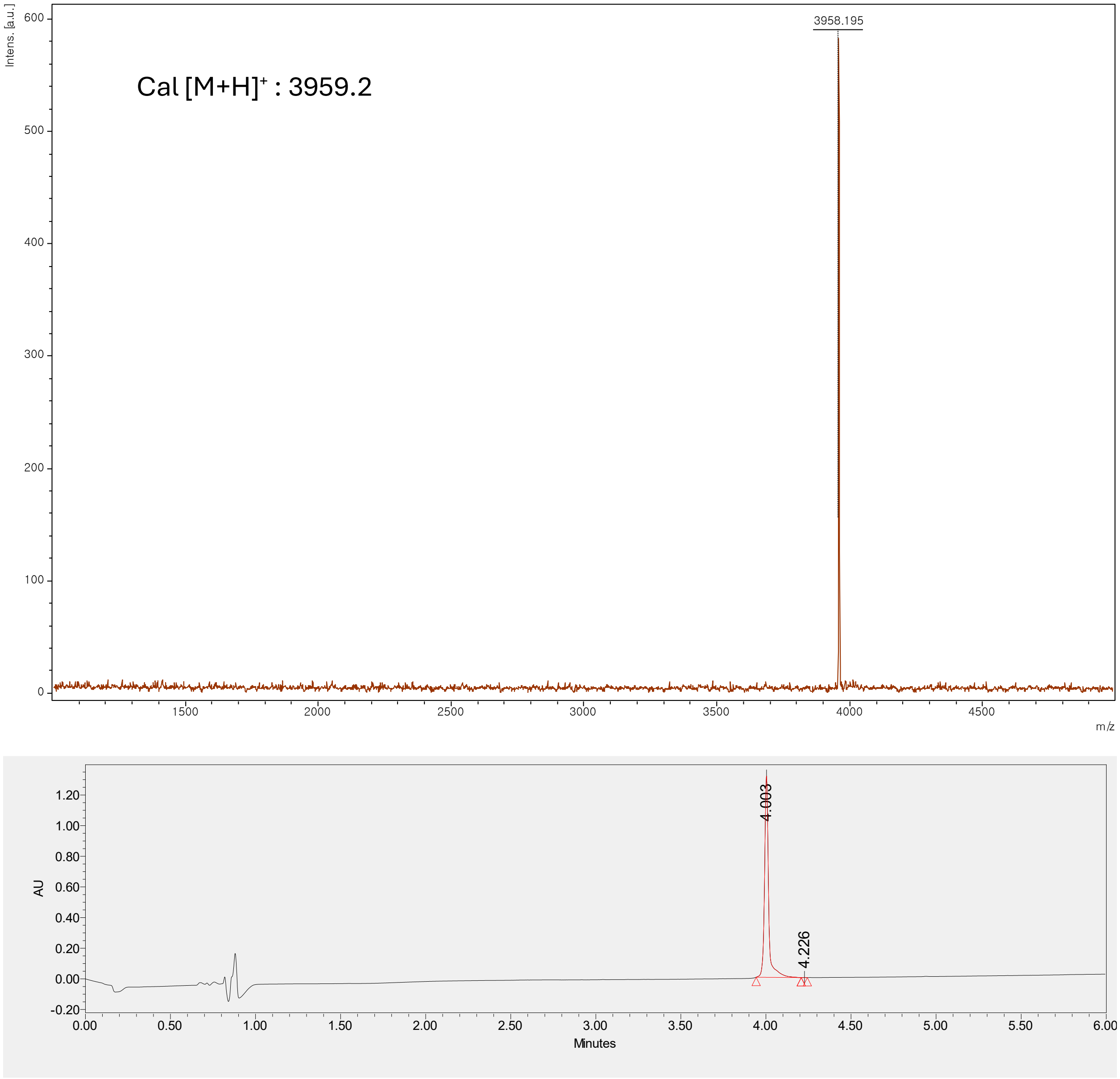

MALDI-TOF-MS (top) and UPLC (bottom) analysis of the ABL peptide. UPLC chromatogram at 220 nm was obtained with a ACQUITY UPLC BEH C18 column (2.1mm X 100mm) eluted with a linear gradient of 10-90% acetonitrile in water (0.1% TFA) applied over 6 min at a flow rate of 0.3 mL/min. Purity >95%.

SPT ; (β1A)VSEIQ(β2L)LHD KGKSIQDLRR RFFXHHLXAE XHTAEI-NH_2
(X: (S,S)-aminocyclopentanecarboxylic acid)

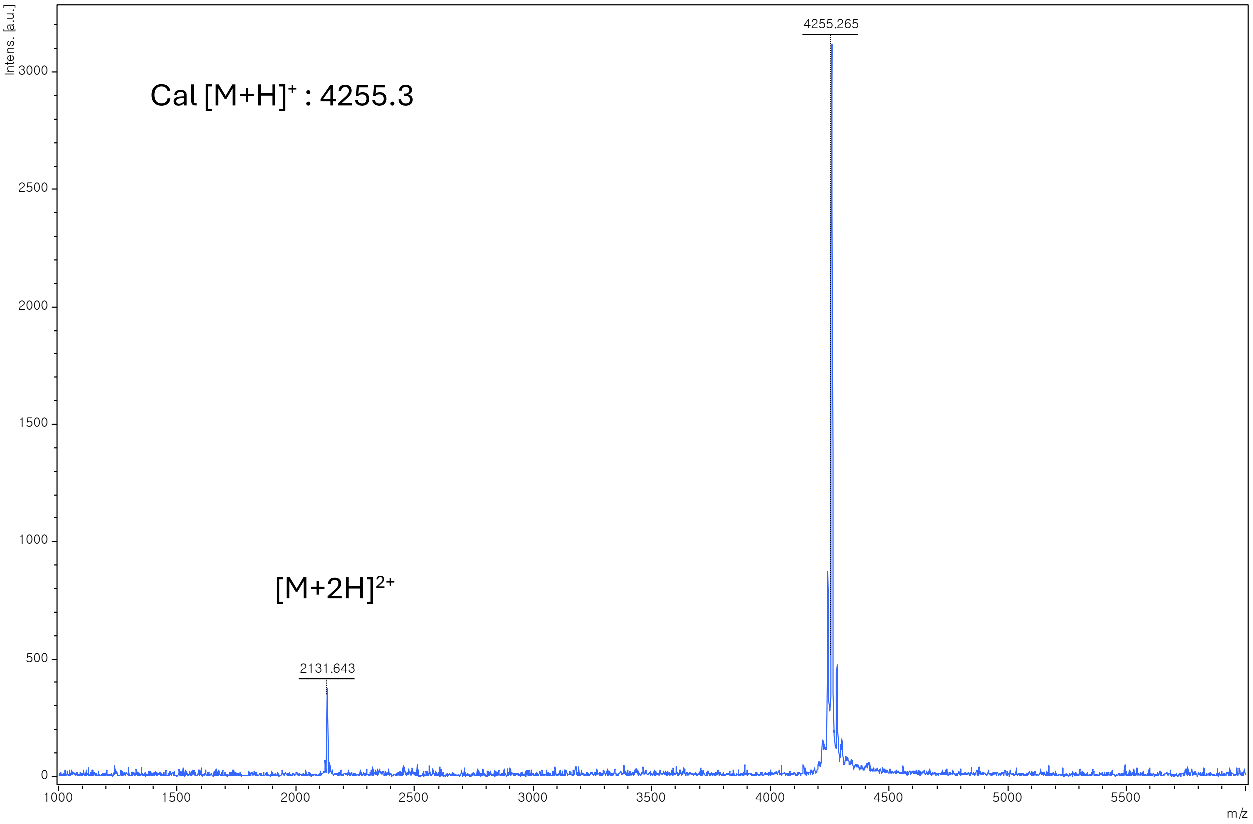

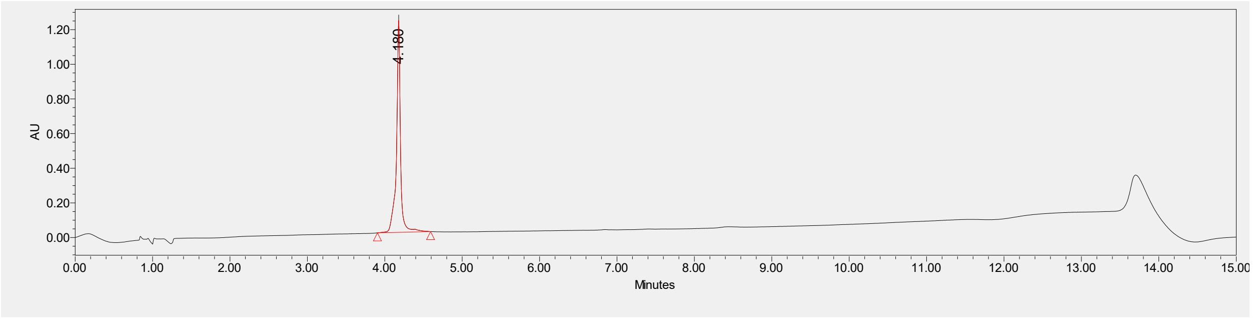

MALDI-TOF-MS (top) and UPLC (bottom) analysis of the SPT peptide. UPLC chromatogram at 220 nm was obtained with a ACQUITY UPLC BEH C18 column (2.1mm X 100mm) eluted with a linear gradient of 10-90% acetonitrile in water (0.1% TFA) applied over 10 min at a flow rate of 0.3 mL/min. Purity >95%.
